# The Bigger Fish: A Comparison of Meta-Learning QSAR Models on Low-Resourced Aquatic Toxicity Regression Tasks

**DOI:** 10.1021/acs.est.3c00334

**Published:** 2023-06-14

**Authors:** Thalea Schlender, Markus Viljanen, Jan N. van Rijn, Felix Mohr, Willie JGM. Peijnenburg, Holger H. Hoos, Emiel Rorije, Albert Wong

**Affiliations:** † Leiden Institute of Advanced Computer Science, Leiden University, Leiden 2333 CA, The Netherlands; ‡ National Institute for Public Health and the Environment (RIVM), Bilthoven 3720 BA, The Netherlands; § Universidad de La Sabana, Chía 250001, Colombia; ∥ Institute of Environmental Sciences, Leiden University, Leiden 2333 CC, The Netherlands; ⊥ Chair for AI Methodology, RWTH Aaachen University, Aachen 52056, Germany; # Department of Computer Science, The University of British Columbia, Vancouver V6T 1Z4, Canada

**Keywords:** QSAR, ecotoxicology, aquatic ecosystem, meta-learning, multi-task learning, learning curves

## Abstract

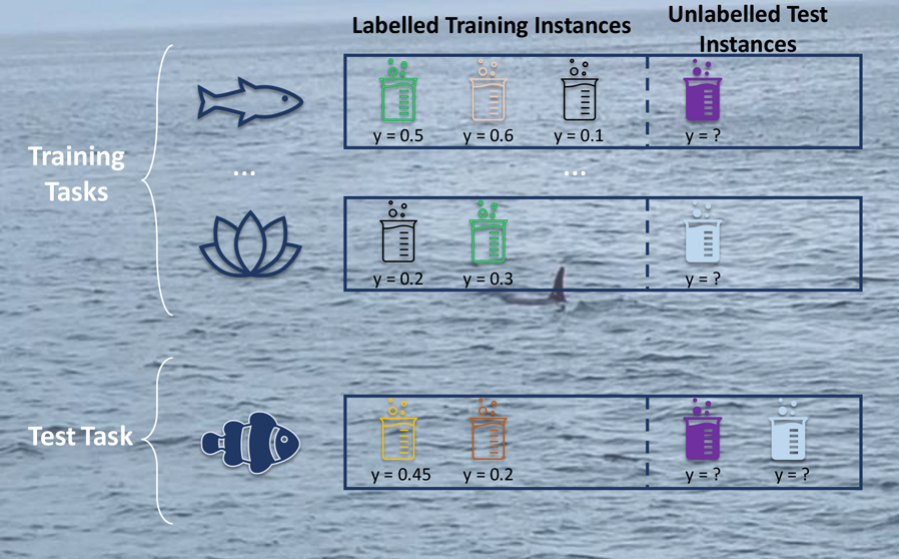

Toxicological information as needed for risk assessments of chemical compounds is often sparse. Unfortunately, gathering new toxicological information experimentally often involves animal testing. Simulated alternatives, e.g., quantitative structure–activity relationship (QSAR) models, are preferred to infer the toxicity of new compounds. Aquatic toxicity data collections consist of many related tasks—each predicting the toxicity of new compounds on a given species. Since many of these tasks are inherently low-resource, i.e., involve few associated compounds, this is challenging. Meta-learning is a subfield of artificial intelligence that can lead to more accurate models by enabling the utilization of information across tasks. In our work, we benchmark various state-of-the-art meta-learning techniques for building QSAR models, focusing on knowledge sharing between species. Specifically, we employ and compare transformational machine learning, model-agnostic meta-learning, fine-tuning, and multi-task models. Our experiments show that established knowledge-sharing techniques outperform single-task approaches. We recommend the use of multi-task random forest models for aquatic toxicity modeling, which matched or exceeded the performance of other approaches and robustly produced good results in the low-resource settings we studied. This model functions on a species level, predicting toxicity for multiple species across various phyla, with flexible exposure duration and on a large chemical applicability domain.

## Abstract 2

In a bid to reduce animal experiments, we analyze the performance of meta-learning quantitative structure−activity relationship regression models on sparse aquatic ecotoxicity datasets. Further, the impact of the amount of data to build these models is investigated.

## Introduction

With the advent of machine learning, the field of Cheminformatics has flourished by using data science techniques on physical–chemical problems. One such problem is the modeling of the bioactivity related to molecular compounds. Known as quantitative structure–activity relationship (QSAR) modeling, the field aims to reduce the need for *in vivo*—in organism—and *in vitro*—in test tube—experiments *via* cost-effective *in silico* simulated approaches. The research in this field has been motivated for decades by the aim of reducing experiments that are expensive in terms of life, cost, and time (see, e.g., Cherkasov et al.^1^).

QSAR models relate chemical structures to their biological activity in a given target domain, from full organisms to specific proteins and even to specific genes. The biological activities that QSAR models aim to predict, are manifold and domain-specific. Toxicity can be measured by the impact a compound has on for instance the mortality, reproduction, mobility, or growth of certain species. Our work specifically addresses the toxicity causing mortality in aquatic species. The prediction of aquatic toxicity as a biological activity has its prevalent use in risk assessment for environmental protection. With the increasing amount of industrial chemicals being used and developed, the European Union Regulation for the Registration, Evaluation, Authorisation and Restriction of Chemical Substances (REACH) requires an investigation into the aquatic toxicity of a chemical released into the environment, for instance through QSAR models.^2^ Due to this regulation, there is a strong need for better-performing aquatic toxicity QSAR models that predict the toxicity of chemicals on various aquatic species such as water flees (so-called *Daphnia*), algae, and fish.

One of the simplest aquatic toxicity models is ECOSAR, proposed by the United States Environmental Protection Agency (USEPA)—a regulatory model that uses a linear relationship between chemicals and their toxicity based on the octanol–water coefficient of the chemical. Based on building different linear regressions on groups of chemicals, ECOSAR is a nonspecies-specific tool for aquatic toxicity. Unfortunately, large safety factors need to be added to the predictions for their use in risk assessment.^3^ With the rise of machine learning, aquatic toxicity models, like other branches of QSAR modeling, have started using machine learning models built for singular tasks, such as USEPA’s Toxicity Estimation Software Tool (T.E.S.T.) developed for three species representing fish, *daphnia*, and algae,^4^ and the more enhanced Vega^5^ toolboxes. For an extensive comparison, we refer the reader to the following overview paper by Zhou et al.^6^

Various extensions to regulatory QSAR models have been proposed. Wu and Wei^7^ applied multi-task learning to a toxicity context by using four toxicity tasks. Alternatively, Lunghini et al.^8^ proposed to build a model for toxicity prediction of fish, *daphnids*, and algae, respectively, associating all assays only with the high-level category, such that the species of an assay cannot be determined anymore. Their model was shown to outperform ECOSAR, T.E.S.T., and Vega on a previously unseen industrial set of toxicity data. In contrast to these high-level models, Singh et al.^3^ propose a model that is trained on a single given species but can extrapolate to a different species in different classes. Recent research has also evaluated the use of graphical features for compounds.^9,10^

Further, Gajewicz-Skretna et al.^11^ reported in a study for classifying aquatic toxicity that models built on a local chemical compound space performed better than ones for large chemical spaces, although they agree with the added value of large models. As such, Sheffield and Judson^12^ built an ensemble learner on a species level, that also aims of building a generally applicable model by restricting their input data as little as possible. Their original work, however, only predicts the toxicity of fish, whereas we expand their approach in our work by predicting across different fish, *daphnia*, and algae. Recognizing the importance of modeling aquatic toxicity across chemicals and species, other work has looked into modeling across species.^13−18^ The challenge of building generally applicable models across species lies in the extreme sparsity of tests between chemicals and species. This suggests that knowledge-sharing techniques may be useful.

To enable knowledge sharing across data sets, the scientific community has developed methods commonly referred to as *meta-learning*.^19^ Whereas traditional machine learning models typically require an abundance of labeled data, meta-learning attempts to address this issue by asking *how to learn to learn tasks?* For this, meta-learning borrows intuition from how humans learn and solve problems. Instead of learning each task independently and anew, humans approach each challenge with prior knowledge.^19,20^ With the success of transfer learning techniques in natural language processing or image analysis, its potential use in QSAR modeling has been recognized.^21,22^ We believe the use of these techniques could be beneficial in utilizing and predicting the many low-resource tasks inherent to aquatic toxicity. Therefore, we investigate several state-of-the-art knowledge-sharing approaches to QSAR modeling and apply these methods to a species-level aquatic toxicity model for multiple species across different phyla with flexible exposure duration. Specifically, we employ multi-task models, fine-tuning, model-agnostic meta-learning, and transformational machine-learning models. Additionally, for purposes of comparison, we consider several baseline methods.

The first approach is multi-task learning, where multiple tasks are learnt jointly using a single predictive model, enabling that model to utilize knowledge across tasks. Erhan et al.^23^ first used a multi-task neural network in their work on collaborative filtering. Dahl et al.^24^ utilized multi-task learning to predict both biochemical (in test tubes) and cell type (in cell cultures) assays. Ramsundar et al.^25^ predicted binary biological activity using “massively” multi-task neural networks built on over 200 tasks with over 40 million experimental values and varying end points. Sadawi et al.^26^ used multi-task learning *via* random forest models previously shown to be effective in single-task cases^27^ on a subset of the ChEMBL data set collection.^28^ Following this literature, we utilize a multi-task random forest, a neural network (two architectures), as well as a stacked ensemble.

We further use fine-tuning models, which, in order to learn an internal representation across tasks, use all tasks to train a model. Then, finally, the model is fine-tuned on a specific test task. By considering all single tasks, model-agnostic meta-learning captures the knowledge across tasks by learning a good initial model representation. This is done in such a way that a model can be efficiently optimized for each task. Nguyen et al.^10^ applied both approaches with graph neural networks to a subset of the ChEMBL data set collection.^28^ Additionally, model-agnostic meta-learning (MAML) is a technique where good initialization weights for a neural network are learned based on which weights can be easily optimized on related tasks.^29^ We use both fine-tuning as well as MAML in our benchmark.

More recently, Olier et al.^30^ proposed a transformational machine learning approach, which takes inspiration from multi-task learning, transfer learning and ensemble learning. The approach aims to learn multi-task-specific compound representations. This representation shares knowledge between all tasks, by encapsulating the general consensus on biological activity. We utilize two proposed versions of this method in our study.

In this article, we aim to model the toxicity of many aquatic species individually in a generally applicable model, which makes no restrictive assumptions on its chemical input. Considering recent research on meta-learning in QSAR modeling, 10 models (consisting of state-of-the-art methods from the previous categories and baseline methods) representing recent developments are adapted and applied for aquatic toxicity prediction. *Via* a data set collection gathered from ECOTOX, consisting of 24 816 assays, 351 separate species, and 2674 chemicals, we carry out a general comparison of the QSAR models with internal and external validation. We also simulate low-resource situations by artificially down-sampling the data sets to few assays per species or few species to share knowledge between. We compare single-species models and multi-species models and assess the benefit of using meta-learning techniques. Finally, we provide useful knowledge to future QSAR developers by investigating the impact of low-resourced situations on the modeling techniques, and we recommend QSAR models to use for aquatic toxicity. All our results are made publicly available *via* a Git repository.^31^

## Problem Statement

This section elaborates on the problem of predicting aquatic toxicity tackled in our work and addresses the domain-specific OECD test guidelines that are used to generate the toxicity data used in ecotoxicological risk assessment, and that therefore guide the QSAR model development.

### Aquatic Toxicity Problem

With the aim of reducing animal testing, *in silico* tools should be able to predict the toxicity of a compound after a specific exposure duration and for the species that it is tested on. Representing the aquatic ecosystem, regulatory tools may as a minimum provide toxicity levels for three representative groups: “acute fish toxicity”, “acute *daphnid* toxicity”, and “alga toxicity”.^3,32^

We aim to build generally applicable QSAR models that predict the toxicity of chemicals across phyla on a species level using training data of all species. Any meta-learning approach should therefore determine which data from related species should be used in modeling the toxicity of compounds on a target species. Our QSAR models are aimed at having a large applicability domain for compounds; they should be able to produce reasonably accurate predictions across a large variety of chemicals. Many QSAR problems are simplified into binary classification tasks, predicting whether a specific compound is toxic or not *via* manually chosen thresholds. In contrast, our work builds regression models, which directly predict the real-valued concentration of a chemical at which 50% of a given species dies—the LC50 (Lethal Concentration 50)

Furthermore, we predict toxicities across variable exposure durations. Our model thus predicts acute and chronic toxicities for species across phyla, leveraging more data across different exposure duration while training the models. Moreover, a model with adaptable exposure duration on the species level could in theory also be used for modeling species sensitivity distributions, which relate the concentration of a compound to the percentage of aquatic species (in a given ecosystem) that will be affected by that concentration.^33^

**Figure 1:**
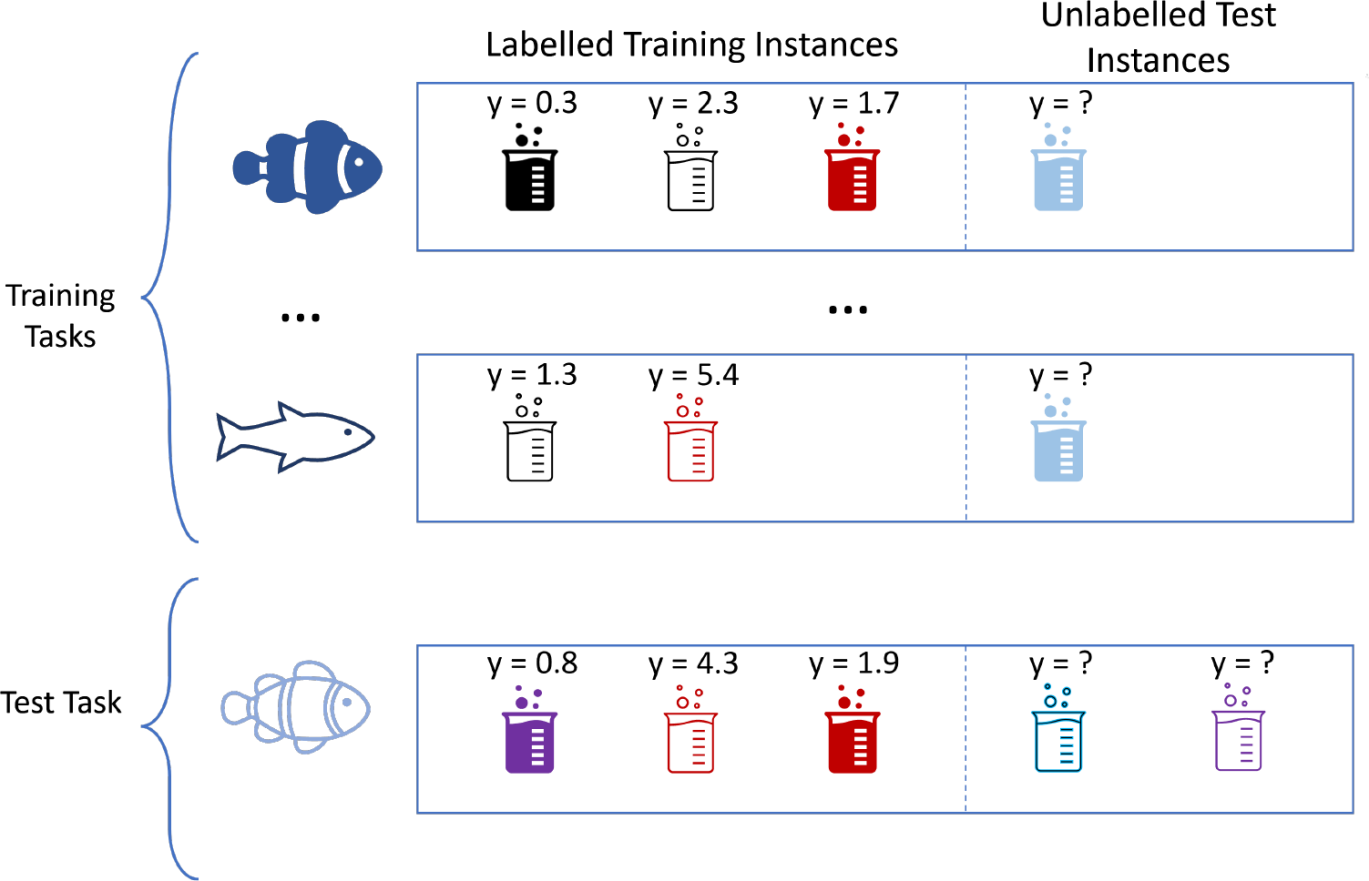
Aquatic toxicity QSAR tasks: The setup of the individual aquatic species tasks. The image shows how the tasks can be used for meta-learning: using the data in the training tasks to utilize additional data for the test task. Meta-learning methods can differ in the way these utilize the training task data. Different colored beakers refer to different chemicals, whereas *Y* represents the measured toxicity effect the chemical has on the species.

To build our aquatic toxicity models, we make use of the fact that QSAR tasks have very similar structures. The issue of aquatic toxicity prediction is split into many (often sparse) tasks: each task refers to a unique target species for which the effect is to be measured (see Figure 1). For each species, several toxicity responses have been measured; these data provide the basis for our machine-learning approach.

While we aim to learn across tasks, the problem setup is defined in such a way that the unlabeled test instances (chemicals) for which we want to predict the LC50 values have not been observed for any of the other species before. This poses a more challenging learning problem, where the learning algorithm has to generalize across structural properties of the molecule. This problem setup has the practical implication that we can predict the LC50 values for newly identified chemicals that have never before been tested on a given target species.

In summary, the proposed models are formally solving the following problem: given a set of chemicals *C* = *C*_train_ ∪ *C*_test_, where *C*_train_ ∩ *C*_test_ = Ø, a compound *c* ∈ *C*_train_, a duration  and a target species *s* ∈ *S*, predict the lethal concentration of a new compound *c*_new_ ∈ *C*_test_ for 50% of *s* ∈ *S* after time duration *d*. Each compound is represented by a molecular embedding and physical-chemical features, whereas for each target species, taxonomical information on its phylum and class group is available.

### OECD Validation Principles

With the increased relevance of QSAR models in the REACH legislation, the need for validated QSAR models of high quality has grown. Addressing this, the OECD principles^32^ present requirements that QSAR models fit for regulatory applications should adhere to. Although our work does not aim to present a model for regulatory purposes but rather aims to inform future development, we address these principles here.

First, to ensure that researchers can assess the potential use of a validated QSAR model, a well-defined end point should be specified. In our work, we address end points in the category of ecological effects, which are included in the end points needed for regulatory assessment.^32^ Specifically, we address the LC50 for most species, as well as the EC50 solely for immobilization of *daphnids* (as this is generally assumed to be a proxy for death). These end points are addressed in a ‘general (Q)SAR model(s) based upon a common toxic effect’^32^ of aquatic species, where the toxic effect refers to death.

To define when a QSAR model may validly be employed, any QSAR model should include a description of the domain of applicability defined in the chemical structure space. This domain should be determined systematically to ensure that a model is not forced to extrapolate into unintended domains and is ideally defined prior to building a training set. Our work, however, addresses the issue that QSAR models are used outside of their applicability domain for low-resource data sets, for which there are insufficient resources for building a single-task model. Hence, we deliberately aim to develop a generally applicable model on given data sets by including different experimental durations and all applicable chemicals. Thereby, we accept the higher uncertainty of the predictions that are possibly out of the domain of applicability.

It is important to note that the training set of a QSAR model always induces a domain of applicability.^32^ Although measuring the domain of applicability is left as future work, it is interesting to note that toxicological data sets have natural biases. Under the REACH program, for instance, chemicals of over 1-ton of production volume need to be registered with toxicological information.^34^ Hence, data sets include biased information on chemicals that are produced at higher volumes, whereas chemicals under the threshold avoid testing, although their acute toxicity may be more concerning.^34^

Further, validated QSAR models need to be reproducible and transparent. To address this, we describe all employed algorithms, data sets, and chemical descriptors and make these publicly available *via* a Git repository.^31^ In our work, black box models, specifically neural network models, are employed that are not transparent but are permitted *via* the OECD guidance document. While there are clear benefits in having transparent and explainable models for some tasks, for other tasks, achieving the highest possible accuracy is more important, which justifies these black box models.

Finally, the performance of a QSAR model must be measured and validated soundly, paying special attention to robustness and predictive capacity. To assess the stability of predictions, we build partial models *via* cross-validation.^32^ The predictive capacity of our model is seen by its performance when extrapolating to an external held-out test set. All models are exclusively evaluated on the real-world challenge of predicting the toxicity of *previously unseen* chemicals, i.e., chemicals not used for training.

## Data

This section presents the data set used to develop our QSAR aquatic toxicity models, of which the preprocessed version is available in our Git repository.^31^ The ECOTOXicology Knowledgebase is a source for locating single chemical toxicity data for aquatic life, terrestrial plants, and wildlife, which is maintained by the USEPA.^35^

**Figure 2:**
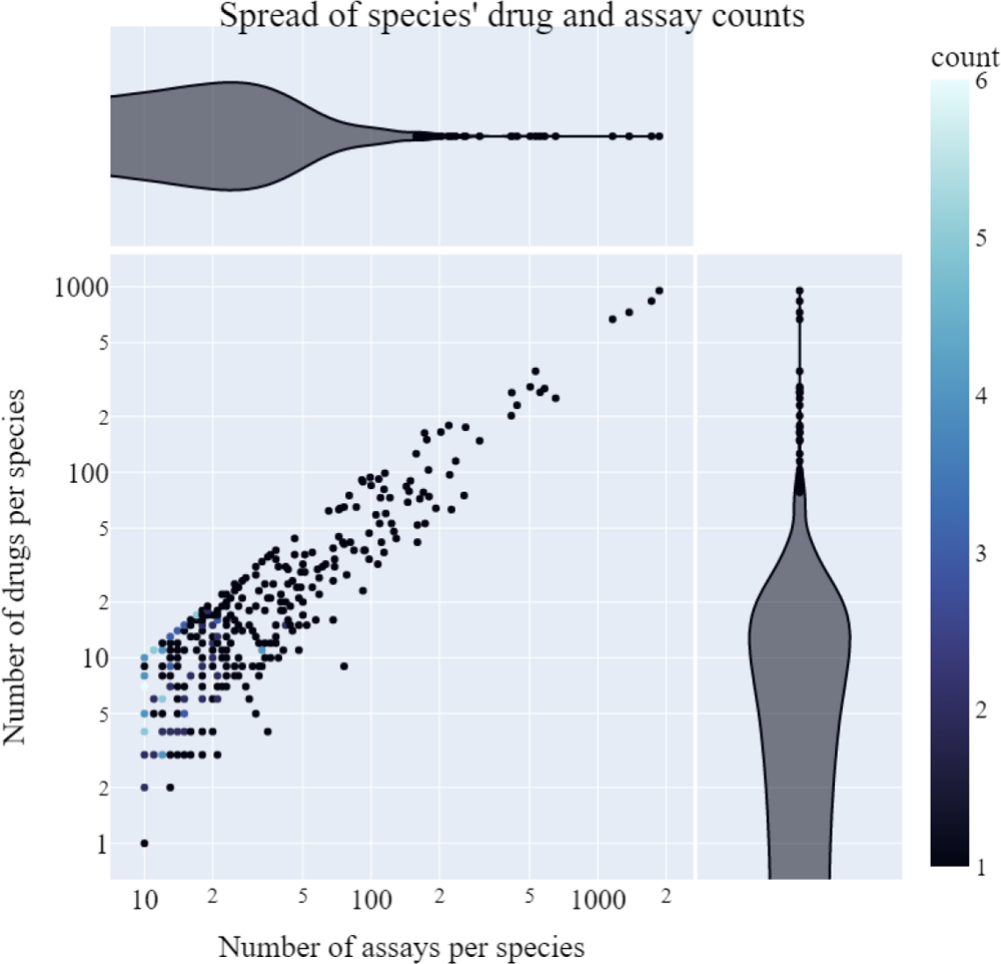
Number of assays and drugs per species. Both axes are on a log-scale.

Using the ECOTOX data as integrated in the OECD QSAR toolbox,^36^ a subselection of the entire database was created for modeling purposes. The final data set used for modeling contained 24 816 aquatic toxicity values (LC50) altogether, for 351 different aquatic species and 2 674 chemicals. Species are described only by their taxonomic position in classes and phyla, whereas chemicals have more descriptive features. The data is sparse, as many species have few chemicals tested on them (see Figure 2).

For our purpose, we have selected all experimental data that was represented as LC50 in the database, i.e., those concentrations giving 50% mortality at the end of the (indicated) test duration.

We kept LC50 read-outs for all test durations. Experiments performed under the same experimental conditions (same chemical, same species, same test duration) multiple times are averaged into one result using the geometric mean, as suggested by the REACH guidance document.^32^ Therefore, in the end, only one LC50 value was generated for any specific combination of chemical, species and test duration. This was considered necessary, as otherwise, some chemicals/species/duration combinations would be overrepresented and thus bias model training. We do note that, by combining multiple toxicity targets for the same experiment, the intertest variability is no longer fully captured and noise is reduced.^37^ Further, as aquatic toxicity values have been gathered over decades in various laboratories, causing variation among experimental values, Lunghini et al.^8^ reported that the ecotoxicological data set qualities heavily impact model performance—a concern also found in other work.^38,39^

### End Point

The toxicity end point—the target variable—to be predicted by our models is the concentration of a chemical needed to trigger a certain toxic effect; here, we have selected 50% mortality (LC50, lethal concentration 50%), on one specific aquatic species and after a specific test duration.

The LC50 values are standardized to  units where possible and dropped wherever not. Due to the spread of the LC50 target, we predict the real-valued log_10_(LC50). End points that indicate bounds (more than, less than, and in between) are disregarded. Higher bounds are due to detection limits of the toxicity experiments when no more of the substance can be dissolved into the water or when it is not practically useful to test with higher concentrations. The data with bounded LC50 values could serve as a very useful validation set for toxicity models.

### Preprocessing

Each database entry contains the concentration of a specific toxicity end point (in our case LC50), which corresponds to a unique combination of species, chemical, and duration. Each of the 351 species is grouped into taxonomies *via* 20 classes and 9 phyla. With the large majority of species belonging to either the Chordata or Arthropoda phylum, this data set is well-suited for predicting the end points needed for chemical regulation.^32^ As the toxicity is to be predicted on a species level, any subspecies of a species were combined into one species *via* their empirical mean.

It was ensured that the chemicals are uniquely identified *via* their SMILES (Simplified Molecular-Input Entry-System) representation. The SMILES were examined to ensure that chemicals not suited for modeling were removed. In this process, SMILES referring to inorganic chemicals (metals or metal salts) and metallo-organic chemicals were excluded. The presence of metals or metal salts is often responsible for the majority of the observed toxicity. Other chemicals that could not be represented by a single SMILES (e.g., mixtures or natural extracts) were also omitted. To ensure that the SMILES representation is consistent for all chemicals, Kekulé SMILES are used, as produced by the Open-source QSAR-ready chemical structure standardization workflow.^40^ The consistent SMILES representation ensures that all chemical descriptors and fingerprints are derived in the same fashion—regardless of how the original SMILES was created (e.g., the SMILES produced by the OECD QSAR Toolbox).

Although it is common to specify one experiment type (and one exposure duration) to use for modeling, our work aims to build a large applicability domain model, enabling the methods to learn across various duration times. Thus, similar to the work of Sheffield and Judson,^12^ all experimental setups are included in the data set and are defined by their duration. With this, short-term (acute) and long-term (chronic) toxicity can be modeled together. As acute and chronic periods vary for each species, the duration is a real-valued feature. Duration values are converted into days wherever possible and disregarded wherever a duration is not specified. In light of building a generally applicable model with few restrictions, no outlier removal was performed.

### Chemical Descriptors

The chemicals are described *via* chemical fingerprints and relevant physical–chemical properties. Fingerprints are embeddings that aim to capture two-dimensional chemical structures. Our work uses circular fingerprints called extended connectivity fingerprints (ECFP), which were specifically designed for QSAR modeling.^41^ Our work uses the original 1024-bit binary ECFP4 fingerprints, which aim to capture precise atom environment substructural features with a radius of 2.^41^ The fingerprints are calculated from their SMILES representation using the open-source RdKit.^42^

As certain physical–chemical attributes may also yield important information on a molecule, relevant physical–chemical attributes were gathered from PaDEL.^43^ The attributes gathered were suggested by a domain expert and include constitutional and hydrophobic attributes. We performed simple feature selection, as well as added missing value indicators, in case PaDEL did not have the values for a given chemical.

Finally, the structural properties included are counts of atom types, rings, hydrogen bond donors, acceptors, as well as the molar refractivity, polarizability, ionization energy, and topological polar surface area of the molecule.

Attributes that are expected to be specifically related to aquatic toxicity are the logarithm of the octanol–water partition coefficient, log  *K*_OW_ or log *P*, the octanol/air partition coefficient *K*_OA_, and the pH-dependent octanol–water distribution coefficient, log*D9*5.5 and log*D*7.4, in addition to the vapor pressure and the water solubility of a molecule.

## Methodology

In this section, the QSAR solutions we considered are elaborated further. We put additional care into optimizing the hyperparameters of each method, which is detailed in the Supporting Information.^44^ The solutions were implemented using Scikit-learn,^45^ Pytorch,^46^ and deepChem.^47^

### Single-Task Models

The single-task models approach each data set individually without using any knowledge of other data sets. As such, they cannot make use of data on other species or their taxonomies.

#### Single-Task Mean

The single-task mean model predicts the mean of training set toxicity values for a given species in training. This is considered a simple baseline: Any model that utilizes additional information should be able to outperform this prediction.

#### Single-Task Random Forest

**Figure 3:**
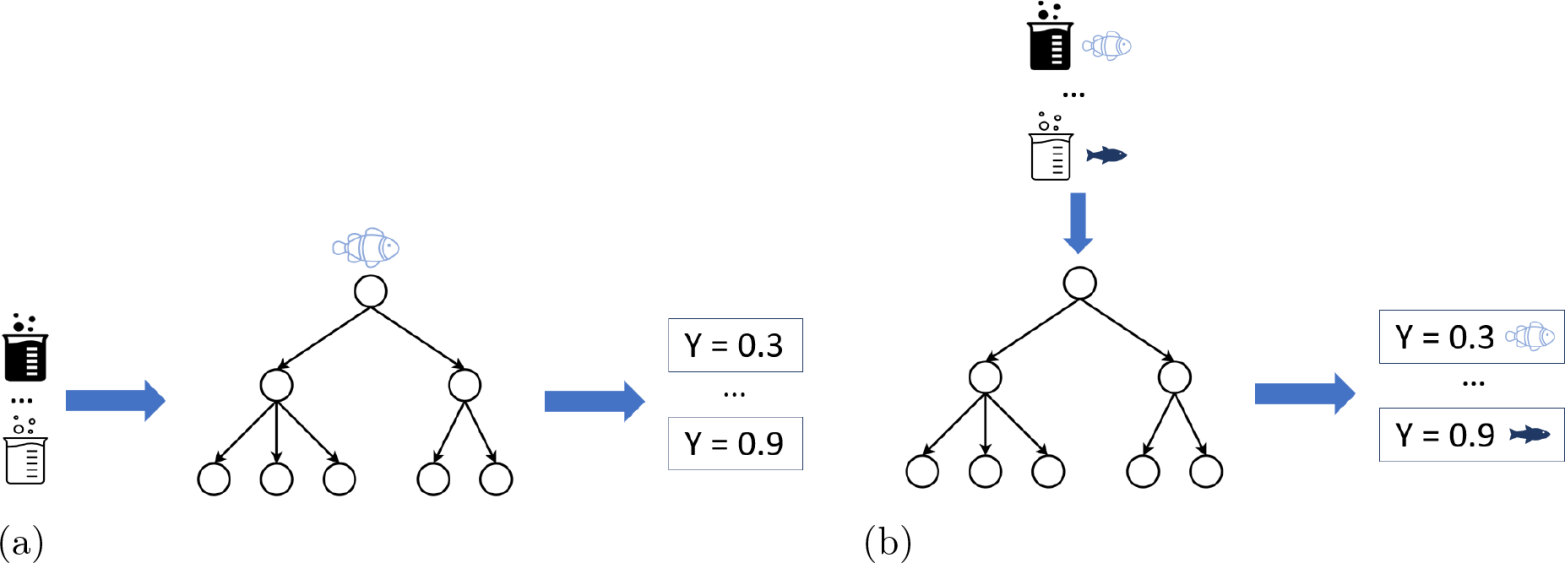
Schematic overview of random forest models. The end point value is represented as ‘Y’; different beakers represent different chemicals.

Random forest models are ensemble models that predict the consensus value across multiple decision trees.^48,49^ Other toxicology studies have found them the best-performing single-task model.^27^ Independent random forest models are fitted for each species using the molecular descriptors and the exposure duration as features, as illustrated in Figure 3a.

### Multi-Task Learning Models

The multi-task learning models learn the separate tasks jointly to share knowledge between them during training. These models can utilize data from different species and make use of features capturing taxonomic information, i.e., species, phyla, and class as categorical variables.

#### Multi-Task Mean

The multi-task mean predicts the mean toxicity value of all species seen in training.

#### Multi-Task Random Forest

In the multi-task random forest model, a single random forest is trained on data from all species, with additional taxonomic information making it possible to give different predictions for different aquatic species (see Figure 3b). The higher-order taxonomy levels may improve the model’s performance if similar species respond similarly (Sadawi et al.).

#### Multi-Task Stacked Ensemble Learner

**Figure 4:**
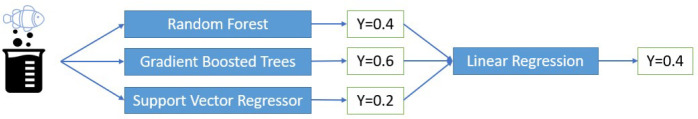
Stacked ensemble learning: base learners are combined into one consensus value. The end point value is represented as ‘Y’.

Sheffield and Judson proposed the stacked ensemble learner, which creates an ensemble from different models by learning how to best combine their predictions. As shown in Figure 4, they used linear regression to combine three base models: support vector regression, gradient boosted trees, and a random forest. All base learners use the molecular descriptors, taxonomic information, and exposure duration.

### Multi-Task Neural Networks

We consider two distinct neural network architectures.

#### One Output Node

**Figure 5:**
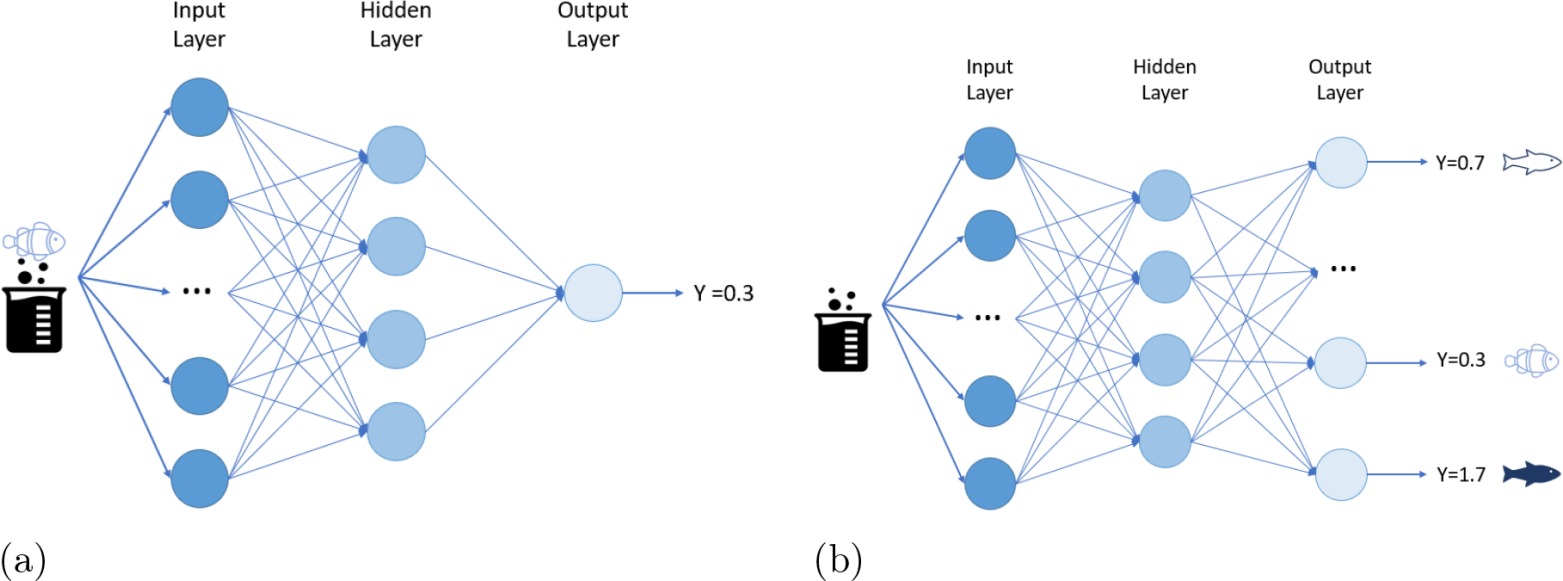
Multi-task neural networks: The end point value is represented as ‘Y’.

The neural network is trained on all of the tasks, but uses only one node in the output layer (see Figure 5a). In addition to chemical descriptors and exposure duration features, including the taxonomic information allows for predicting a different toxicity value for different species. We refer to this model as neural network with one output node, *NN - 1 output*.

#### Multiple Output Nodes

The *multitarget* neural network, *multitarget NN,* predicts the toxicities of all *n* tasks using *n* output nodes (see Figure 5b). This allows the neural network to share the internal feature extraction and representation part embedded in the hidden layers of the neural network, whereas the task-specific dependencies can be captured in the weights toward the task-specific output nodes.

### Transformational Machine Learning

**Figure 6:**
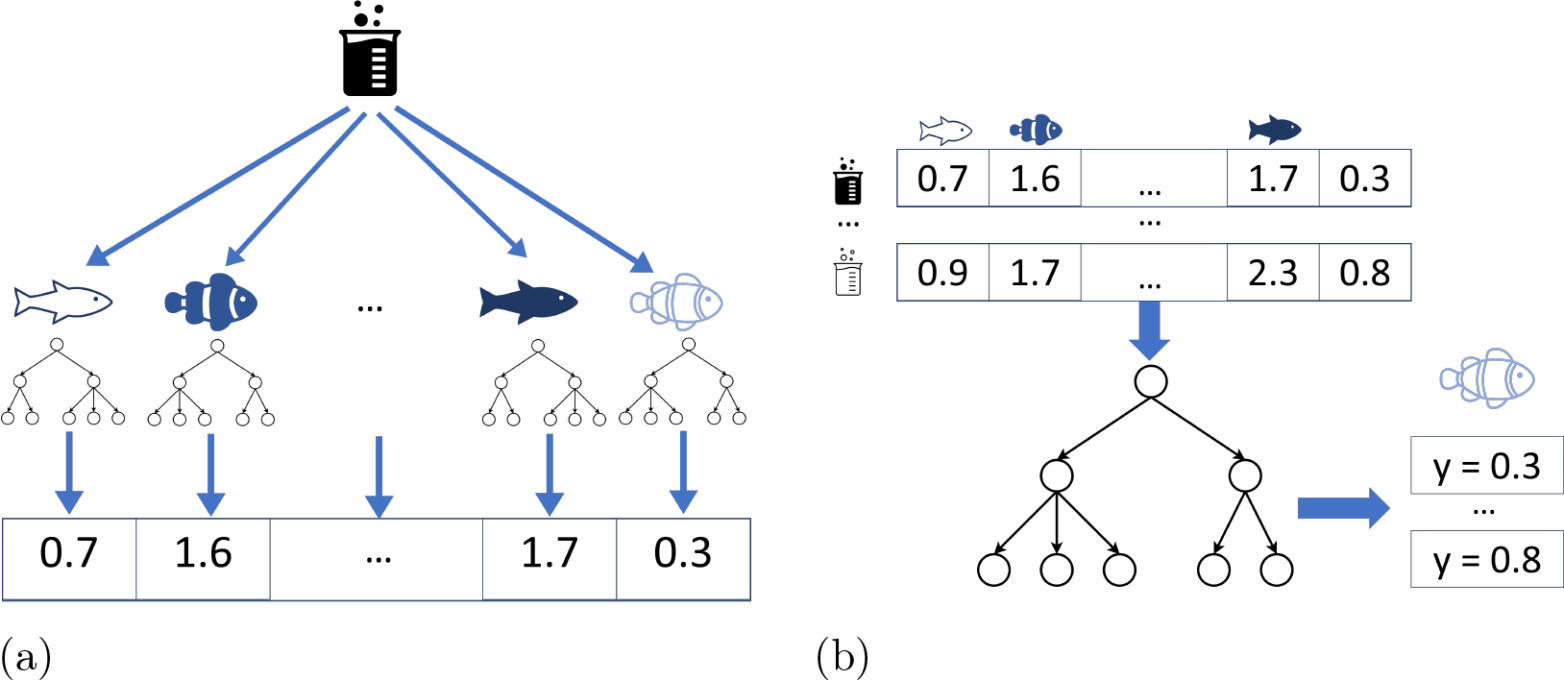
Transformational machine learning.^30^ The end point value is represented as ‘Y’; different beakers represent different chemicals.

Transformational machine learning (TML)^30^ combines aspects of ensemble-, multi-task-, and transfer learning. It can be split into two parts:

1. Create a shared representation of the compound: A single-task random forest is fitted for each target species. Once all single-task models have been built, they predict the toxicity of a specific compound for all species, as shown in Figure 6a. These predictions are then placed in a vector, which will be our representation for the compound.
2. Build final single-task models: A single-task random forest model is fitted for all target species, respectively, but the input features are now the representations from Step 1 (see Figure 6b). By training a single-task random forest for a given species, the model can learn to use the general consensus over similar species in the vector.

We use two variations of this model: the one described above (TML) and one aggregating this prediction with the single-task random forest model trained in the first step (TML Stacked).

### Fine-Tuning

Fine-tuning techniques are a simple way to perform transfer learning with neural networks.^50^ First, a neural network is trained on all tasks to extract knowledge from the input features and build an internal representation; then, (a selection of) the weights are adapted to the final task. As suggested in the literature, a neural network is trained on all species, before the weights of all layers except for the head are frozen, and the head of the network is trained on the given aquatic species. To emphasize that we follow the literature, we refer to this method as *finetuning top*.

### Model Agnostic Meta-Learning

**Figure 7:**
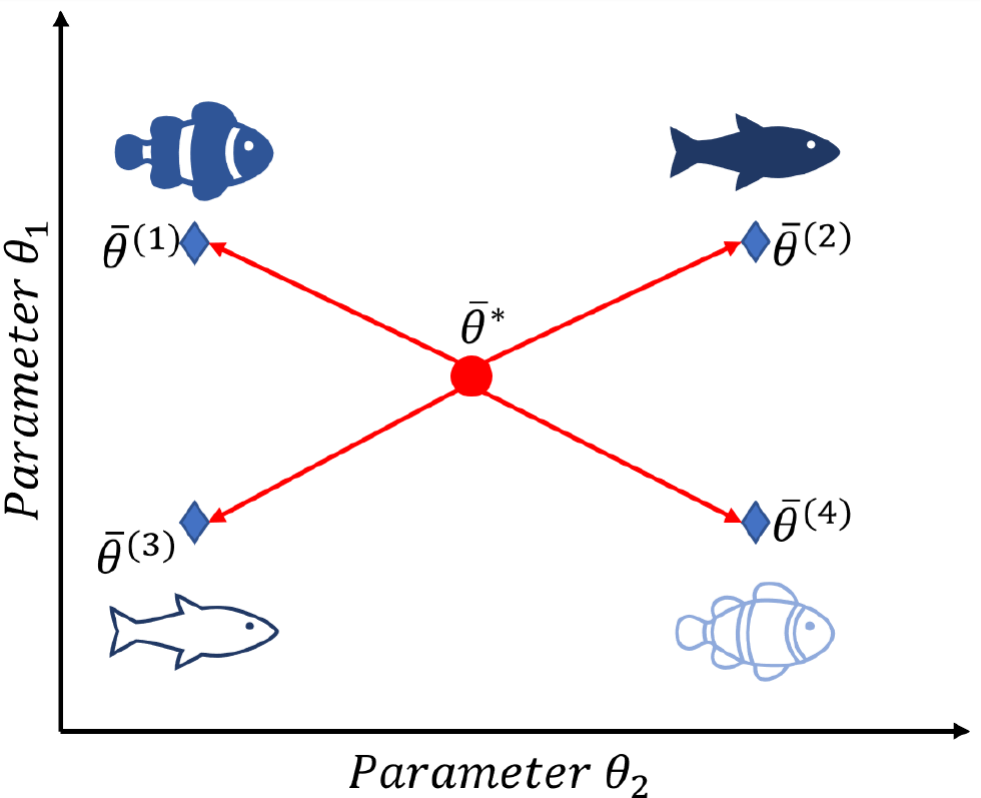
Intuition behind MAML:^29^ Let the model used have initialization parameter vector . The blue points show the optimal configuration of initialization parameters  for specific species tasks 1–4. MAML aims to find , such that the optimal configuration for each task can be reached equally fast.^51^

Model agnostic meta-learning (MAML)^29^ is a model-agnostic transfer learning technique. We use it with a neural network. The initialization weights of a standard neural network are random values and require a substantial amount of training data to adapt for a given task. MAML aims to encapsulate knowledge from related tasks into good initialization parameters. It observes which weights worked well for related tasks to suggest initial weights that can be quickly adapted to a new task. In contrast to fine-tuning, which adapts weights found optimal for all tasks to a single-task, MAML aims to find initialization weights that allow for quick adapting to all tasks,^51^ as illustrated in Figure 7.

## Experiments

**Figure 8:**
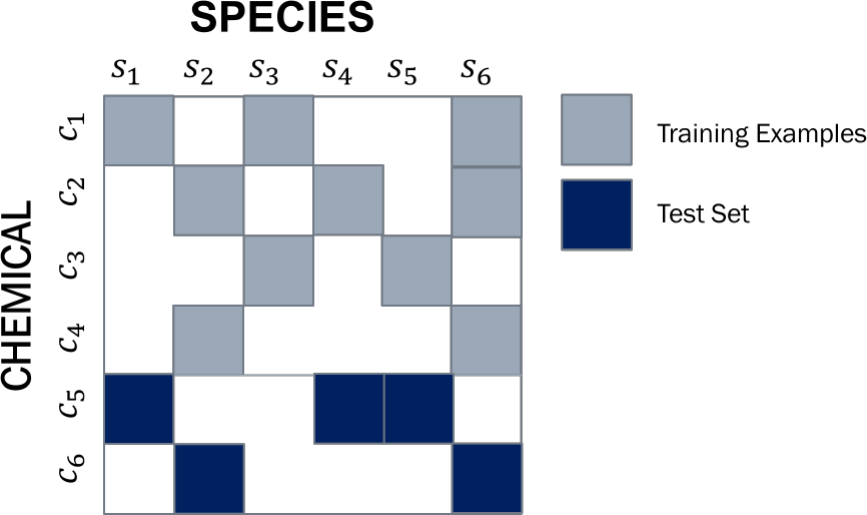
Training vs testing data: The rows represent chemicals, whereas the columns represent the study species. Our training and testing data consist of disjoint subsets of chemicals.

In the following, we examine the prediction quality of QSAR algorithms on new chemical compounds for which no observations (assays) were used during training. For our experiments, we therefore partition the ECOTOX data set uniformly at random into *training chemicals*, which are used for training our models, and *test chemicals*, which are used for assessing their quality. This scheme is illustrated in Figure 8, with test assays shown in dark blue; duration times are omitted for simplicity.

We address the following research questions:

R1 What is the average prediction performance of the previously discussed (hyperparameter optimized) QSAR algorithms on previously unseen chemicals?

R2 How does the performance of the models (both single-task and multi-task) increase when exposed to more data from the target species?

R3 How does the performance of the models increase when more data from other species (to learn across data sets) is available?

Prediction performance is measured in terms of the root mean squared error (RMSE)
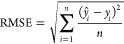
1where  is the *predicted* and *y*_*i*_ is the *true* label for the *i*-th out of the *n* test assays over which the metric is being computed. The performance error is calculated per species/chemical/fold and averaged over all species/chemicals/folds.

In addition, a Friedman test^52^ is used according to the suggestion by Demšar^53^ to determine the statistical significance of performance differences among multiple algorithms. We test whether and to what extent any pair of algorithms statistically differ in performance; we refer to our Supporting Information for details.

### Average Prediction Performance of QSAR Algorithms

To properly assess the prediction performance of the QSAR algorithms, we proceeded as follows. According to the previously described splitting scheme, we allocated 80% of the chemicals for training and 20% for testing; we call the respective portions of the ECOTOX data set the *internal* and *external* folds. Then, two types of experiments were conducted. The first assesses the predictive capacity of each hyperparameter-optimized QSAR algorithm trained on the internal fold when extrapolating to the external fold.

The second experiment assesses the stability of each QSAR algorithm *via* cross-validation. To this end, the *chemicals* contained in the internal fold were partitioned into five disjoint and equally sized subfolds (each one containing 20% of the chemicals). We built five hyperparameter-optimized partial models that exclude a subfold from the training set to subsequently predict.

This process was repeated three times with different partitions, yielding 15 estimates for each QSAR algorithm.

#### Results

**Figure 9:**
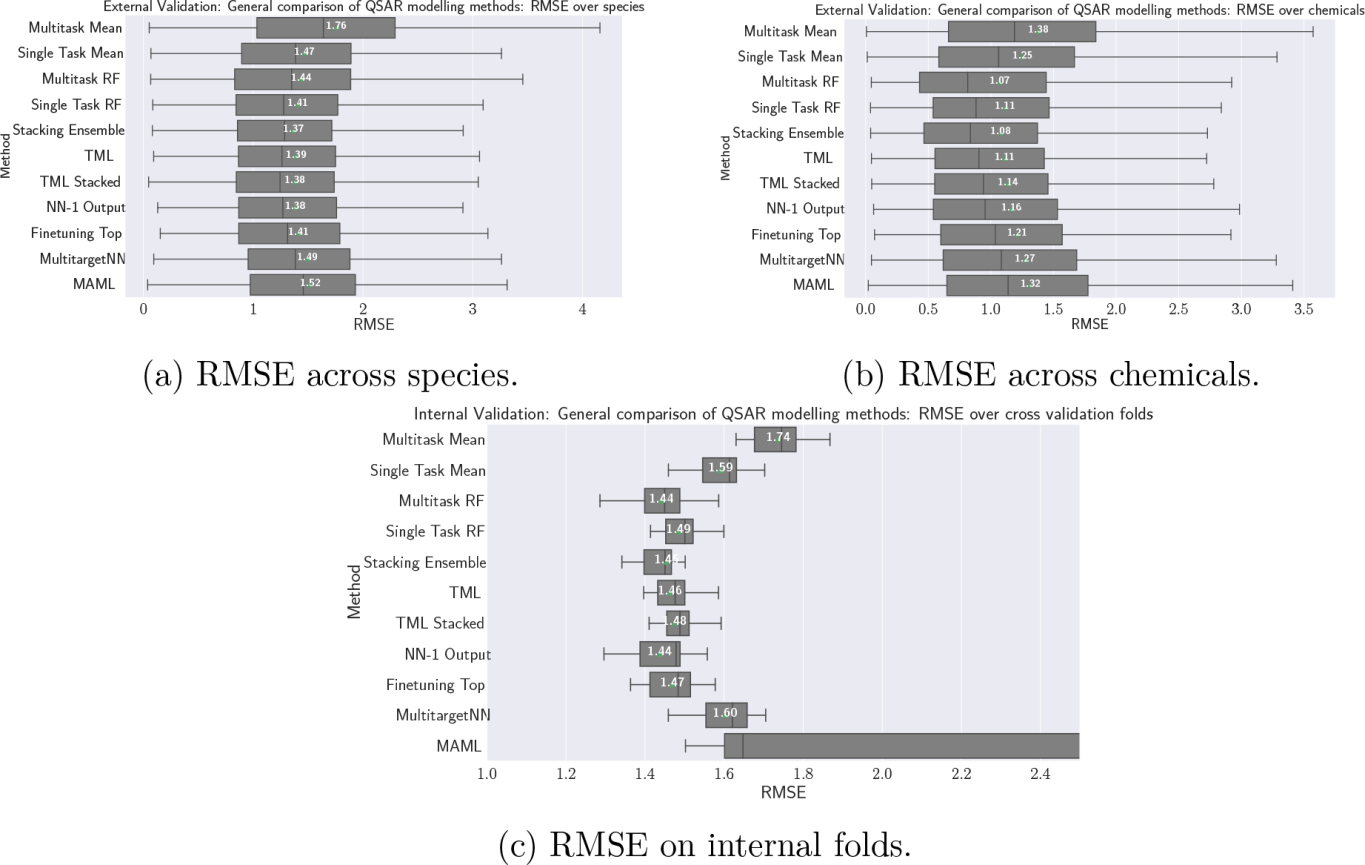
Comparison of prediction performances (RMSE) of different algorithms. The labeled green marker indicates the mean of the observed performance values, whereas the line represents the median.

Figure 9 shows the performances of the QSAR algorithms, with the results aggregated across species as well as chemicals. The plots in Figure 9a,b show the RMSE of the hyperparameter-optimized algorithms on the external test fold, once aggregated across species and once across (test) chemicals. Note that these are the results of the same experiment, but the way of grouping the predictions (either according to species or according to chemicals) affects the weight of the individual predictions, therefore also resulting in different performance estimates. These numbers, therefore, give an unbiased estimate of the out-of-sample prediction performance of the models.

The best-performing methods are the multi-task random forest and the stacked ensembling method. Aggregating over chemicals, their mean test RMSEs are 1.07 and 1.08, respectively. The differences between the techniques are mostly statistically significant; we refer to the supplement for details.

Predictions with an RMSE of less than 1 are within a factor 10 of the original LC50 value (before applying the log-scale), which makes such models useful for various applications when certain error margins are applied, including risk assessments of regulators; we refer to the supplement for a derivation.

The results in plot 9b show that the *median* RMSE of several methods is indeed below 1, so at least for a significant portion of chemicals, the methods can be considered to work acceptably or even very well: The two previously mentioned techniques are the only ones with a 25% quantile below 0.5.

Plot 9c summarizes, for each of the algorithms, the 15 validation results of the internal hyperparameter optimization procedure; it hence reflects the stability of the performances (narrow boxplots indicate high stability of the procedure and thus that the results in plots 9a and 9b are close to the true average results). Plot 9c underlines that these results can be considered largely stable. For most methods, the performance only changes marginally with the chemicals selected for training. The only exception is MAML, which is too unstable for use but does not perform competitively under any observed condition anyway.

### Prediction Performance as a Function of Number of Assays

#### Experiment Setup

Addressing research question R2, we now study the effect of more data on the target species. For this, we utilize learning curves for each of the algorithms.^54^ First, the union of internal and external data was split uniformly at random, using 90% of the chemicals for training and 10% for testing. We then identified all species for which at least 128 training assays were available (with the goal to form reasonably long useful learning curves). Specifically, a species is then included if it has 128 training assays or data points that involve the species, a number of chemicals from the training chemicals, and one or more exposure durations. The 35 species that satisfied this criterion are called the *study species*. For the remaining species (with few assays), training assays were moved into an *auxiliary data set*, and test assays were removed entirely from the data set. Finally, learning curves in the form of RMSE as a function of the number of training assays (per species) were computed.

We built the learning curves as follows: For each *anchor* (training set size) , all of the QSAR algorithms were trained using the training assays and then the RMSE was computed on the test assays. The number of assays used at *s* was *s* for each model in a single-task learner and 35 · *s* (with *s* samples from each of the 35 study species) for multi-task learners. The assays used at previous anchors were included in the following anchors, e.g., the assays used at the anchor utilizing five assays were also used for training at the following anchors utilizing 8, 11, and 16 assays and so on. To reduce the effect of selecting the assays in a certain order, we built not only one but *three* such curves with different assays order and then averaged.

**Figure 10:**
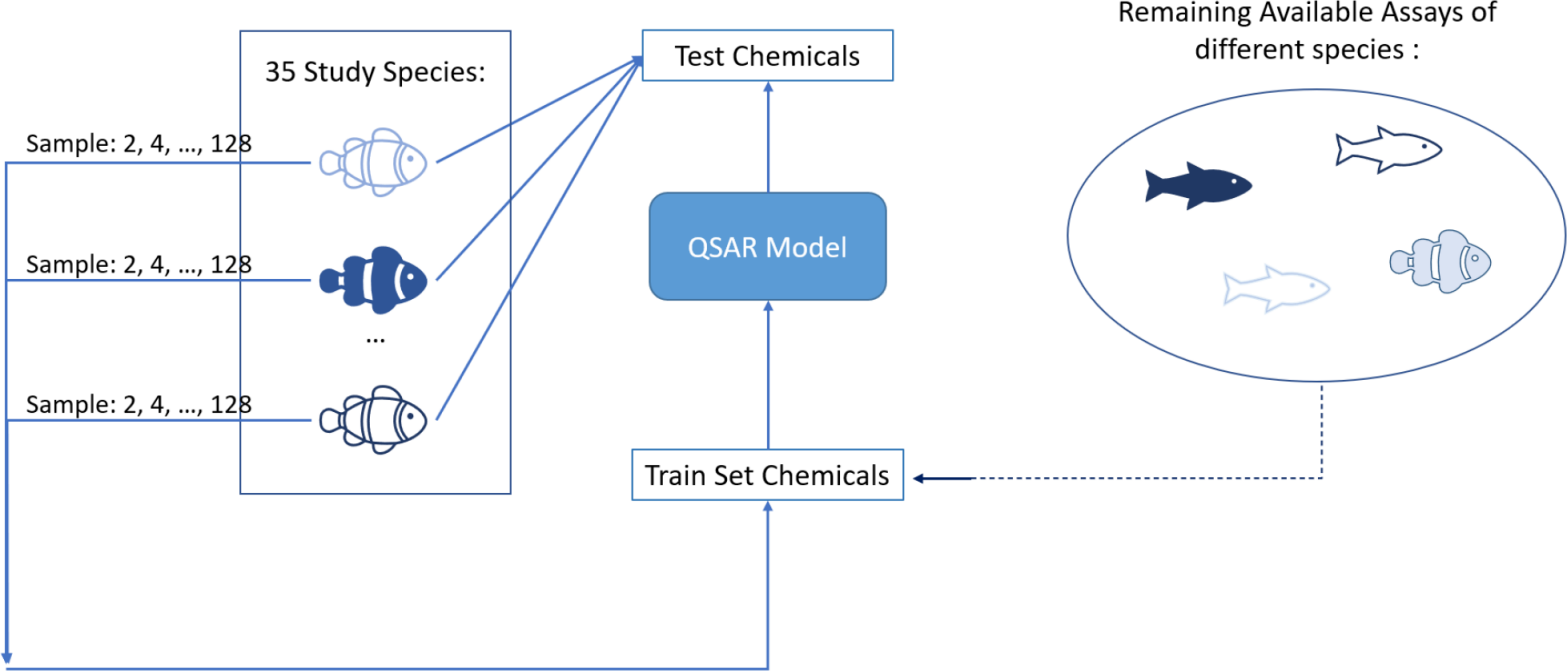
Schematic view of the experimental setup. Using 35 study species in our train and test set, a harsh low-resource situation is simulated, with the training set containing only the down-sampled species, whereas the second scenario adds the remaining assays from other species to the training set too.

With this, we now elaborate on how the models perform when more data is being presented in two different settings. In the first case, only assays from the 35 study species were used for training. In the second case, *all* the (10 200) assays from the auxiliary data set (remaining species) were used in addition during training at each anchor. Figure 10 shows a schematic overview of both setups.

#### Results

**Figure 11:**
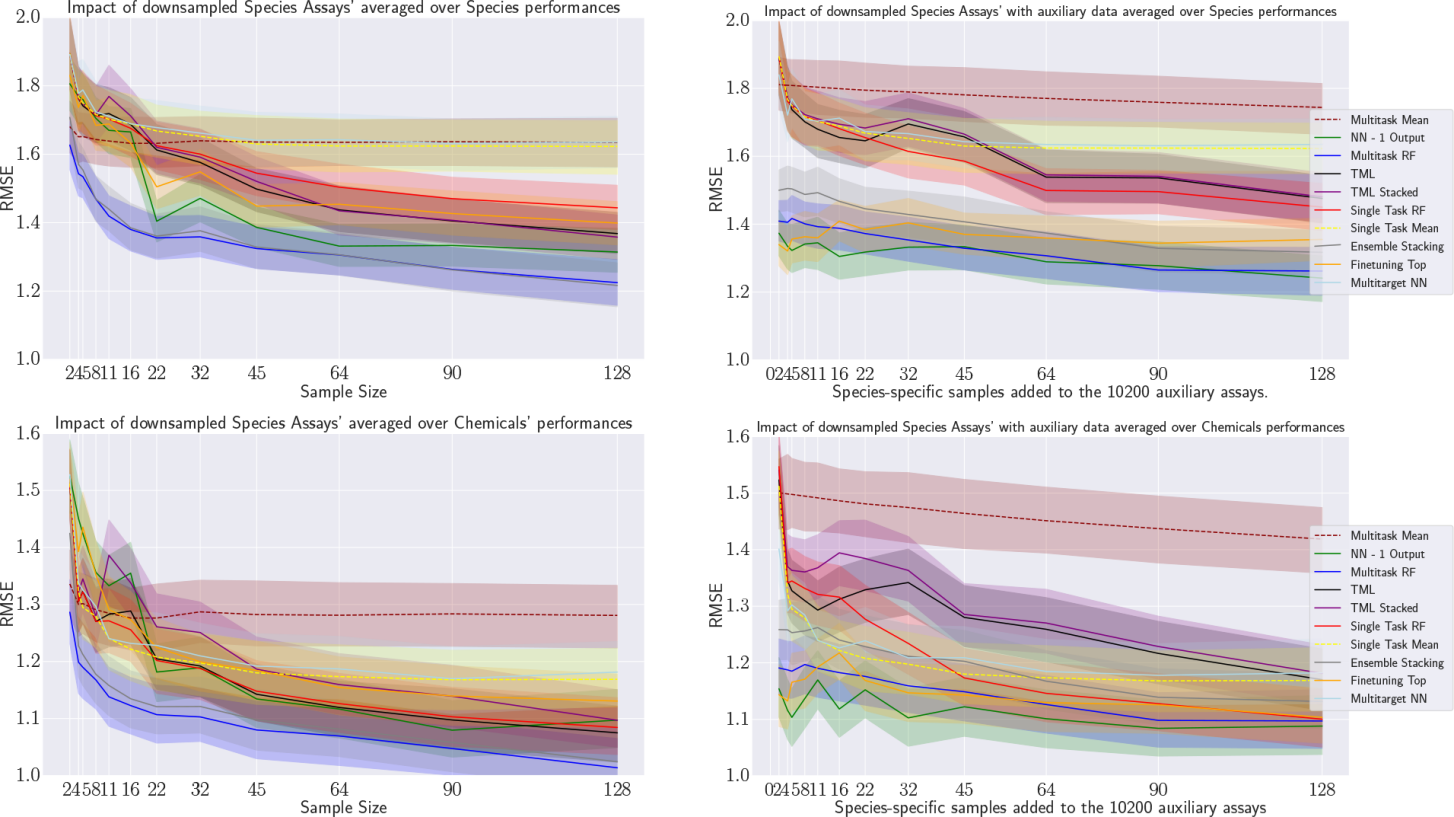
Learning curves showing the effect of downsampling the study species without (left) and with (right) auxiliary species available for training. Once grouped over species (top row) and once over chemicals (bottom row).

The plots in Figure 11 show learning curves without (left) and with (right) auxiliary data available for training. In the top row, the RMSE is computed for each species, and the curves aggregate the species-wise errors, whereas the bottom row aggregates over chemicals.

Lines show mean values and shaded areas the 90% confidence bands computed from 1000 bootstrap samples.

Looking at the left plots, it can be seen that the advantage of the two multi-task methods—i.e., the multi-task random forest and ensemble stacking—are rather independent of the number of assays used for training. The curves are constantly below the others, so these two algorithms are constantly the best choices, no matter how many training examples are being used.

An even more important observation is that all curves are significantly dropping throughout the entire interval under study, including the 128 anchor. A first implication is that all the QSAR algorithms indeed exhibit an ability to *learn* to predict LC50 from the ECOTOX data (otherwise curves would plateau immediately). Second, the fact that curves keep dropping significantly at anchor 128 suggests that it might be fairly possible to predict LC50 even with a *satisfactory* RMSE below 1.0 if more assays were available.

The right plots suggest that auxiliary assays are advantageous if and only if very few species-specific assays are available. The general observation across all multi-task learning algorithms is that the learning curves start off better but decrease less steeply. The first implication is that, if less than roughly 20 training assays are available for a species, it is likely that the random forest or stacking ensemble can benefit from the auxiliary assays. In those common, low-resource cases, using a neural network (with or without finetuning) will do better than learning only with the assays from the study species alone. However, the second implication is that, if more assays are available for the study species, it seems better to ignore auxiliary assays, since they seem to slow down the learning process. This holds at least for random forests and stacking ensembles, both of which show better performance at the 128 anchor when no auxiliary species are being used. Additionally, TML is dependent on all of its single-task models’ performances, as well as the length of its representation here. If so many assays are available and if a neural network is used, the auxiliary species should be used, and the network should not be fine-tuned on the study species. However, given the slope of the learning curves, with 128 assays or more, it seems best to just use a random forest or stacking ensemble without auxiliary assays.

### Prediction Performance as a Function of Number of Species

In this learning curve experiment, we investigate research question R3: to what extent does the number of species included in the training sets affect the performance of multi-task models?

First, the union of internal and external data was split as outlined previously, using 75% of the chemicals for training and 25% for testing. Second, we identified all the species for which there are at least three chemicals among the test assays.

The resulting 180 species are the *study species*; this set happened to be disjoint from the 35 previous study species. Third, to ensure a reasonable number of training instances, among the remaining species, we identified the ones with at least 64 training assays. The resulting 64 species (coincidentally, there were 64 species as well) are called the *auxiliary species*. The assays for all the other species were discarded.

**Figure 12:**
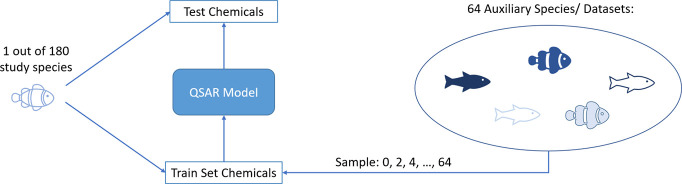
Study setup: Iterating over 180 study species, a study species with its training and test set, is selected. Sampling 0, 2, 4···, 64 auxiliary species into the training set, a new QSAR model is built. With this, the impact of adding more species to aid in learning a study species is shown.

To determine the learning curves, we proceeded as follows. First, the number of assays per auxiliary species was down-sampled to 64. This was done, because the number of samples per auxiliary species varied from 64 to over 1 000, so a change in a performance curve could have been attributed to the fact that many data samples were added to the training set and not primarily to the addition of additional species to infer knowledge from. Second, a random permutation of the auxiliary species was created. Third, for each study species, the RMSE for each QSAR algorithm was computed on the test assays when using the respective training assays and the 64 training assays from each of the  first auxiliary species, where *n* ∈ {1, 2, ..., 12}, for training. The experiments were repeated over three different pseudorandom number seeds, inducing different down-sampled auxiliary data sets and different permutations of the auxiliary species. The general experimental setup is schematically shown in Figure 12.

#### Results

**Figure 13:**
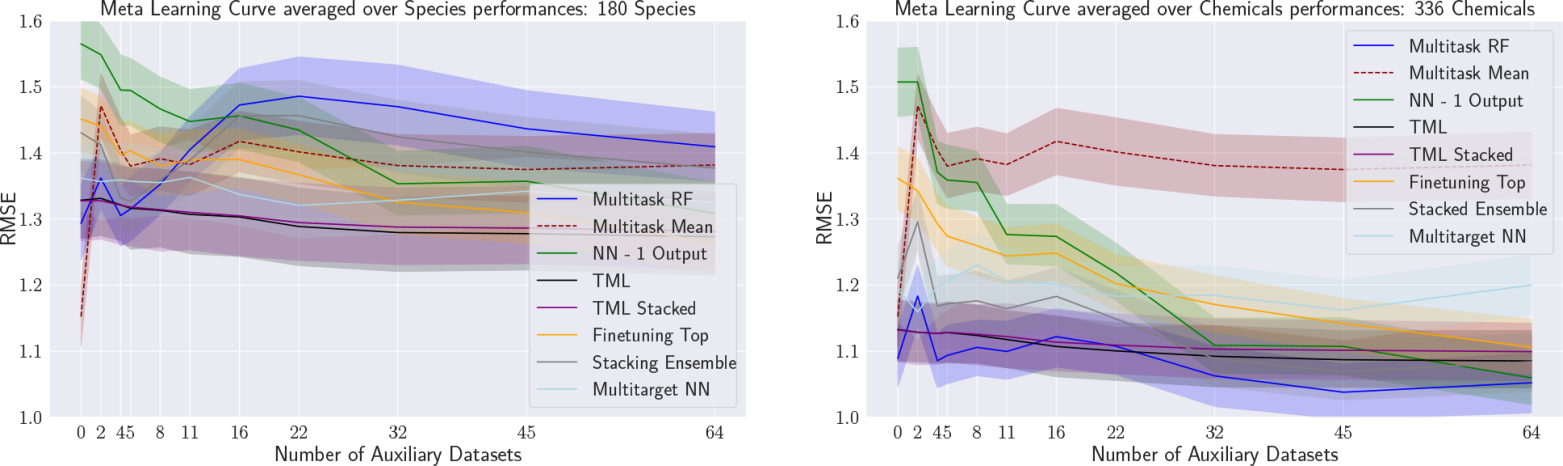
Learning curves showing the effect of adding more auxiliary species to the training set of a study species. Results averaged over species (left) and chemicals (right).

Figure 13 shows the performances, with the left plot aggregating over species and the right over chemicals. Note that single-task models have been omitted, as they do not make use of additional data.

To answer the research question R2, we observe that the benefit of additional training assays from other species is only significantly beneficial for NN with one output unit and for Finetuning-Top. The curves of the other algorithms have a shallow improvement or even partially deteriorate (e.g., multi-task RFs when averaging over species). For these two algorithms, the additional data though does have a rather interesting effect. At the highest anchor (64 additional species), Finetuning-Top achieves the best results when averaging over species, and the NN with one output is not outperformed when averaging over chemicals.

More importantly, both algorithms still show significant learning progress at that point on the curve. In other words, one might conjecture that adding assays from additional species would lead to overall results superior to those of other learners and possibly lead to results below the 1.0 RMSE threshold.

However, these assessments must be viewed with caution. The test matrix for the defined over the test chemicals and the 180 study species is extremely sparse, which has several side effects. First, TML is now working consistently better than on the 35 study species of the previous learning curve even though it only marginally improves with increasing additional training assays from other species. Hence, TML behaves very differently on the species/chemical combinations analyzed in this experiment than in the previous one.

Second, the two previous best models (multi-task random forest and stacking ensemble) also perform very well in this setup when averaging over chemicals but not when averaging over species. This is caused by a single chemical for which prediction qualities are low for most species, and due to the sparsity of test data, this has a high influence when averaging over species but a low influence when averaging over chemicals—in other words, the results are very sensitive to the species/chemical combinations used for training and testing respectively (details can be found in the Supporting Information).

A further hypothesis addressing these differences may be different instance weightings between single- and multi-task models. To achieve a generally good performance, a multi-task model aims to predict the majority of assays well. Due to the large differences in the number of assays with a certain chemical or species, the multi-task model may aim to predict the largest groups of chemicals or species better. A single-task model, however, could concentrate on each species more equally, as a separate model is built for each task. The single-task models optimize for good performance over species, whereas when the models are averaged over chemicals the single-task models are not as good as the multi-task model. Future work should investigate how the choice of evaluation affects the relative order, and further, it may be interesting to experiment with instance weighting explicitly by weighting training instances while building a model.

Overall, the results motivate future work in which the selection of species and chemicals is studied further. Learning across certain more related tasks (species or chemicals), that were more carefully selected, may further benefit model performance. An alternative could be adding more detailed, scaled-task-relatedness measures to replace the categorical species taxonomies.

In the sense of meta-learning, this could motivate a context-based approach, in which the learning algorithm itself is chosen based on the properties of the species and/or the chemicals for which training instances are available or predictions need to be made, as is done in the work of Olier et al.^27^

## Discussion

Our work has addressed modeling LC50 values (mortality rate in 50% of the experiments) of different aquatic species, specifically using a collection of well-known sparse ecotoxicological data sets. To make predictions for species with few assays, we explore the use of different machine-learning techniques to leverage additional data from other species. We pay special attention to addressing domain-specific requirements *via* the OECD principles, and we evaluate the models in a setting where we make predictions for the toxicity of species for a chemical that has not been seen before for any of the other species. This is motivated by the fact that this is the most common use-case of toxicological predictions, which can be readily applied when a new chemical needs to be evaluated.

Based on our experiments, for this problem setting, we advise the use of the multi-task random forest model. Its performance is stable, as seen in the internal validations, and the performance is good on external validations, both averaged over chemicals and species. Furthermore, the multi-task model also performs well in simulated low-resource situations. When looking at the general data sets consisting of all assays, there is no statistical evidence that the multi-task random forest performs better than the single-task random forest. The multi-task random forest model has a lower performance error than its single task version in 54% of unseen chemicals. However, when examining cases, in which there were less than five seen compounds for a species, the multi-task random forest outperforms the single task random forest in 80% of unseen chemicals. Extrapolating onward from the learning curve experiments, the neural network with one output unit seems promising with more assays available.

As we believe that the inclusion of class and phyla information aids the multi-task models, we hypothesize that a continuous distance measure between the species could further enhance these models. Therefore, in future work, different, potentially more easily obtainable measures of target relatedness following the tree-of-life notion could be investigated. Furthermore, our investigation into low-resource situations *via* learning curves has given more insight into individual approaches. A future investigation could evaluate the effect of selecting chemicals and species with more care.

Further work in QSAR modeling should therefore investigate the use of knowledge-sharing techniques. Specifically, future work should also anticipate the need for explainable models, which would add the ability to trace back predictions. These explainable models could lead to more insight into aquatic toxicity, especially when these models can utilize knowledge across species.

To conclude, we successfully present multi-task models on a species level that predict toxicity on flexible exposure duration and a large chemical applicability domain, showing promising results for models with general chemical applicability as well as applicability across phyla. With this research, we hope to not only take a step toward mitigating the need for *in vivo* experiments but also hope to inspire the use of knowledge-sharing approaches for other low-resource QSAR problems.

## Supporting Information Available

Discussions of hyperparameter optimization, connection between rules of thumb on the prediction error, statistical significance between algorithms on the average prediction performance, influence of data sparsity in the learning curve performances, and performance on real low resource tasks, tables of hyperparameter optimization for the single-task and multi-task random forest models, hyperparameter optimization for the stacking ensemble model, hyperparameter optimization for the multi-task neural network with one output node, hyperparameter optimization for the fine-tuning approaches, hyperparameter optimization for MAML, and overview of all features in the gathered dataset, and figures of critical distance plots for statistical differences of mean ranks, comparison of prediction performances (RMSE) of different algorithms, heatmap of prediction errors of the multi-task random forest for a chemical per species, RMSE performances averaged over species for different compound counts seen, and prediction performances on actual low-resource datasets (PDF)
