# Supplementary material for: The Bigger Fish: A Comparison of Meta-Learning QSAR Models on Low-Resourced Aquatic Toxicity Regression Tasks
Source: Environ Sci Technol. 2023 Jun 14;57(46):17818–30. doi: 10.1021/acs.est.3c00334 (PMC10666535; doi:10.1021/acs.est.3c00334)
Supplement: Supplementary file 1 — es3c00334_si_001.pdf [file es3c00334_si_001.pdf]

Supporting Information: The Bigger Fish: A comparison of Meta-Learning QSAR models on low-resourced aquatic toxicity regression tasks

Thalea Schlender,[*,†,‡] Markus Viljanen,[‡] Jan N. van Rijn,[†] Felix Mohr,[¶] Willie JGM. Peijnenburg,[‡,§] Holger H. Hoos,[‖,†,⊥] Emiel Rorije,[‡] and Albert Wong[‡]

†*Leiden Institute of Advanced Computer Science, Leiden University, Leiden, 2333 CA, The Netherlands*

‡*National Institute for Public Health and the Environment (RIVM), Bilthoven, 3720 BA, The Netherlands*

¶*Universidad de La Sabana, Chía, 250001, Colombia*

§*Institute of Environmental Sciences, Leiden University, Leiden, 2333 CC, The Netherlands*

‖*Chair for AI Methodology, RWTH Aaachen University, Aachen, 52056, Germany*

⊥*Department of Computer Science, The University of British Columbia, Vancouver, V6T 1Z4, Canada*

E-mail: thalea.schlender@gmail.com

Contents

List of Figures **S3**

List of Tables **S3**

1 Hyperparameter Optimisation **S4**

1.1 Single-task Random Forest S4

1.2 Multitask Learning Models S5

1.2.1 Multitask Random Forest S5

1.2.2 Multitask Stacked Ensemble Learner S5

1.2.3 Multitask Neural Networks S6

1.3 Transformational Machine Learning S8

1.4 Fine-tuning S8

1.5 Model Agnostic Meta-Learning S8

2 Connection Between Rules of Thumb on the Prediction Error **S10**

3 Statistical Significance between Algorithms on the Average Prediction Performance **S11**

3.1 Statistical tests S11

3.2 Results S12

4 The influence of data sparsity in the learning curve performances **S14**

5 Performance on real low resource tasks **S15**

6 Data **S19**

References **S22**

List of Figures

S1 Critical distance plots for statistical differences of mean ranks in the *internal validation* with a significance value of $\alpha = 0.05$. S13

S2 Comparison of Prediction Performances (RMSE) of different algorithms. The green marker shows the mean of the performances, whereas the line refers to the median performance. S13

S3 Critical distance plots for statistical differences of mean ranks in the *external validation* with a significance value of $\alpha = 0.05$. S14

S4 A heatmap of prediction errors of the multitask random forest for a chemical per species S16

S5 Prediction performances on actual low-resource datasets. S18

List of Tables

S1 Hyperparameter Optimisation for the single-task random forest models. Note that the cross validation folds are always defined on subsets of chemicals. . . S4

S2 Hyperparameter Optimisation for the multitask random forest models. . . . S5

S3 Hyperparameter Optimisation for the stacking ensemble model. S5

S4 Hyperparameter Optimisation for the multitask neural network with one output node. S7

S5 Hyperparameter Optimisation for the multitarget multitask neural network. S8

S6 Hyperparameter Optimisation for the fine-tuning approaches. S9

S7 Hyperparameter Optimisation for MAML. S9

S8 Overview of all features in the gathered dataset. S21

1 Hyperparameter Optimisation

A special focus is placed on the hyperparameter optimisation of each method. All hyperparameter optimisation is evaluated on *unseen* chemicals. Hyperparameter evaluations - where possible - are done via cross-validation, as to obtain a stable estimate of the best parameters.

1.1 Single-task Random Forest

We perform hyperparameter optimisation for the single-task random forest models. The additional costs that occur when hyperparameter optimising all single-task models individually, however, is infeasible in our work. Instead, we find data-driven hyperparameter defaults. For this, we find the hyperparameter configuration that leads to the best average performance across all single-task random forest configurations. The hyperparameter optimisation set up can be seen in Table S1. The cross-validation folds are subsets of chemicals, such that the hyperparameter optimisation is performed on the same use case as the model will be employed in. Using 3 folds here is a trade off between the variance caused by the specific training data and computational cost.

Table S1: Hyperparameter Optimisation for the single-task random forest models. Note that the cross validation folds are always defined on subsets of chemicals.

Single-task random forest

Search Algorithm	Random Search
Search Space	Adapted from Autosklearn[S1]
Iterations	50
Cross Validation Folds	3
Performance criteria	RMSE across all single task models
Performed	At every model build

1.2 Multitask Learning Models

1.2.1 Multitask Random Forest

The hyperparameter optimisation for the multitask random forest model can be seen in Table S2. The procedure is nearly identical to the single-task random forest's hyperparameter optimisation, however, instead of searching for data-driven defaults of the hyperparameters for the many single-task models, we are searching for an optimal hyperparameter configuration for the multitask random forest model here.

Table S2: Hyperparameter Optimisation for the multitask random forest models.

Multitask random forest

Search Algorithm	Random Search
Search Space	Adapted from Autosklearn[S1]
Iterations	50
Cross Validation Folds	3
Performance criteria	RMSE
Performed	At every model build

1.2.2 Multitask Stacked Ensemble Learner

The hyperparameter optimisation set up can be seen in Table S3. Note that the hyperparameter optimisation is done for all three base learners individually.

Table S3: Hyperparameter Optimisation for the stacking ensemble model.

Stacking ensemble

Search Algorithm	Random Search
Search Space	Adapted from Autosklearn[S1]
Iterations	50
Cross Validation Folds	3
Performance criteria	RMSE
Performed	On all three base learners individually at every model build

1.2.3 Multitask Neural Networks

To avoid overfitting on the training set, early stopping is implemented. Essentially, this technique splits the training set into a training and validation set. The neural network is trained on the test set as usual, but the validation set allows us to monitor when the neural network seems to be overfitting. For this, the training error and validation error are observed. Once the validation error starts to rise and has not recovered for a certain number of iterations, training is halted. The number of iterations to wait is called *patience* and is a hyperparameter. The validation set is chosen to be 20% of the training set.

Since this technique uses parts of the training set, and the QSAR problem is naturally low-resourced, the training is repeated on the whole training set to ensure that all of the available data is used. In the second training phase, the neural network is trained for as many epochs as the early stopping phase ran for. Typically, it is also possible to run for the number of epochs minus the patience. The additional training data can, however, benefit from the extra iterations as well. We simply rerun the neural network training for the same amount of epochs.

To optimise a neural network, a good neural architecture needs to be found, before the hyperparameters of the learning algorithm are tuned.

First, a neural architecture needs to be found that works well with the problem to solve. The neural architecture search involves finding how deep and wide the network should be, such that performance can be optimised. For this, the number of layers (to find the network's depth) and the number of nodes per layer (to find the network's width) need to be found. Further, the use of regularisation techniques that are embedded into the network needs to be evaluated. Preventing the model to overfit, the regularisation techniques considered here are dropout[S2] and the addition of batch normalisation.[S3] Dropout is a technique that does not use a fraction of the nodes during training iterations to prevent overfitting. Normalising the output of a layer, batch normalisation scales the output such that the output has a mean of zero and a standard deviation of one.

To perform a neural architecture search, the neural network intelligence (NNI) from Microsoft is used,[S4] as it is compatible with Pytorch,[S5] which is used to implement the neural networks themselves. Using the Tree-structured Parzen estimator (TPE),[S6] the use of 3 or 4 layers is evaluated, in addition to the number of nodes in a layer, the scale of dropout, and the addition of batch normalisation. TPE belongs to the class of sequential-based model optimisation methods, which train independent models. We use 50 iterations of TPE on 5-fold cross-validation, in which no two folds had common molecular compounds. The use of a more sophisticated search method for the neural architecture may provide an advantage over other models. The neural architecture search, however, is run only once and this optimised neural architecture is used in all further neural networks that train on multiple tasks, e.g. fine-tuning.

Table S4: Hyperparameter Optimisation for the multitask neural network with one output node.

Multitask neural network one output node

Search Algorithm	Tree-structured Parzen estimator (TPE)[S6]
Hyperparameters optimised	Learning rate, weight decay, batch size and patience
Iterations	50
Cross Validation Folds	5
Performance criteria	RMSE
Performed	Once

The second area that needs to be optimised are hyperparameters of the optimiser, such that the weights can be learned effectively. For optimising the weights of the network, Adam,[S7] an alternative to the classic stochastic gradient descent is used. Adam has been shown to handle sparse gradients well, a problem which may arise with the sparse binary fingerprint inputs. The hyperparameter optimisation set-up is given in S4. Again, the use of the Tree-structured Parzen estimator (TPE)[S6] for the hyperparameter optimisation may give this method an advantage. The hyperparameter configuration, which is found after performing hyperparameter optimisation once, is used in the fine-tuning methods.

Multitarget multitask neural network As Ramsundar et al.[S8] criticise the variance in multitask QSAR neural network models, they propose a standard adaptable model implemented in DeepChem. The neural architecture found for the neural network with one output node is used, although the output nodes are updated. The hyperparameter optimisation is presented in Table S5.

Table S5: Hyperparameter Optimisation for the multitarget multitask neural network.

**Multitarget multitask neural network**	
Search Algorithm	grid search
Hyperparameters optimised	Learning rate, weight decay
Iterations	16
Cross Validation Folds	5
Performance criteria	RMSE
Performed	Once

1.3 Transformational Machine Learning

The transformational machine learning approaches uses the data-driven hyperparameter defaults found for the single-task random forest models.

1.4 Fine-tuning

The hyperparameters used in the finetuning phase are optimised via the set-up in Table S6, whereas the hyperparameters for the general training are taken from the multitask neural network with one output node.

1.5 Model Agnostic Meta-Learning

MAML has several hyper parameters: the learning rate for the task-specific optimiser, α, the learning rate for the initialisation parameter optimiser, β, the number of adaptation steps the task-specific optimiser trains on a task n and the batch size m of tasks trained before the initialisation weights are updated.

Table S6: Hyperparameter Optimisation for the fine-tuning approaches.

Fine-tuning

Search Algorithm	grid search
Hyperparameters optimised	fine-tuning iterations, learning rate, weight decay
Iterations	48
Cross Validation Folds	2
Performance criteria	RMSE
Performed	Once for both finetuning approaches, respectively

As MAML learns good initialisation weights for a neural network to adapt quickly to a new task, we search for a good default single-task neural architecture. For this, the same set-up for the multitask neural architecture search, see 1.2.3, is employed, which searches different neural architectures using 2 or 3 layers is evaluated, rather than 3-4 layers. The performance criteria used was the averaged performances of all single-task neural networks with the same architecture.

Once the default architecture is found, a brief hyperparameter optimisation is done for MAML in the form of a grid search shown in Table S7. The MAML training procedure uses the same early stopping approach as explained earlier. It uses a patience of 25 iterations, as MAML's training is typically more unstable than directly training simple neural networks. We chose to use one training step for all new tasks to adapt from the initialisation weights from MAML.

Table S7: Hyperparameter Optimisation for MAML.

MAML

Search Algorithm	grid search
Hyperparameters optimised	meta learning rate, learning rate, weight decay
Iterations	34
Cross Validation Folds	5
Performance criteria	RMSE
Performed	Once

2 Connection Between Rules of Thumb on the Prediction Error

In the main paper we stated that an acceptable value of the RMSE would be any value that would not exceed 1. We here quickly describe how this can be derived.

Among community experts, which include authors of this paper, it is common sense that a threshold for acceptable (mean) mistakes on the prediction of LC50 is a *factor* of 10. That is, it should be the case that

$$0.1 \leq \frac{1}{n}\sum_{i=1}^{n}\frac{\hat{y}_i}{y_i} \leq 10,$$

where here we assume that $\hat{y}_i, y_i$ are the predicted and true value of LC50, respectively (here *not* on a log-scale, as opposed to the main paper). Unfortunately, this rule of thumb cannot be directly expressed as an RMSE.

However, it is possible to express this rule of thumb in terms of an RMSE if

1. models operate on the $\log_{10}$ of the actual LC50 values
2. we assume that the ratio $\frac{\hat{y}_i}{y_i}$ is identical across all instances i

While the latter is naturally never the case is practice, one can argue that, for a rule of thumb, the assumption is not too severe. Under these conditions, it holds that there exist some $\hat{y}, y$ so that for all i it holds that $\frac{\hat{y}_i}{y_i} = \frac{\hat{y}}{y}$, which is equivalent to $\log_{10}\hat{y}_i - \log_{10} y_i = \log_{10}\hat{y} - \log_{10} y$.

It then follows that

$$\begin{aligned}
\frac{1}{n}\sum_{i=1}^{n}\frac{\hat{y}_i}{y_i} \leq 10 &\Leftrightarrow \frac{1}{n}\sum_{i=1}^{n}\frac{\hat{y}}{y} \leq 10 \\
&\Leftrightarrow \frac{\hat{y}}{y} \leq 10 \\
&\Leftrightarrow \log_{10}\frac{\hat{y}}{y} \leq 1 \\
&\Leftrightarrow \log_{10}\hat{y} - \log_{10} y \leq 1 \\
&\Leftrightarrow \frac{1}{n}\sum_{i=1}^{n}(\log_{10}\hat{y} - \log_{10} y)^2 \leq 1 \\
&\Leftrightarrow \frac{1}{n}\sum_{i=1}^{n}(\log_{10}\hat{y}_i - \log_{10} y_i)^2 \leq 1 \\
&\Leftrightarrow \sqrt{\frac{1}{n}\sum_{i=1}^{n}(\log_{10}\hat{y}_i - \log_{10} y_i)^2} \leq 1 \\
&\Leftrightarrow RMSE \leq 1
\end{aligned}$$

The other inequality follows just by symmetry since $\frac{1}{n}\sum_{i=1}^{n}\frac{\hat{y}_i}{y_i} \geq 0.1 \Leftrightarrow \frac{1}{n}\sum_{i=1}^{n}\frac{y_i}{\hat{y}_i} \leq 10$, again assuming identity of the quotients and that, within the squared term, the logarithms can be flipped without causing side effects.

3 Statistical Significance between Algorithms on the Average Prediction Performance

3.1 Statistical tests

For further statistical analysis, the rank of each method on each task is computed. That is, the performances (measured by the RMSE metric) on a given task are ranked across models. The model with the lowest RMSE for the given task receives a 1, the second-lowest RMSE receives a 2, whereas the highest RMSE receives the highest number. Ties are resolved by using assigning methods the mean rank between them, such that if two models are tied for

the first rank, both receive 1.5.

To compare the performance of multiple algorithms on multiple datasets to each other statistically, Demšar[S9] suggests using the Friedman test.[S10] The Friedman test assesses the hypothesis that the mean of each population is equal. For comparing multiple algorithms on different tasks, the null hypothesis is that the mean of the ranks of a model (over all tasks) is equal to all other models' rank means. If we can reject this null hypothesis, we can investigate which models' means are significantly different to each other.

Once it has been rejected that the mean of all method's ranks is equal, the Nemenyi test[S11] can be performed to determine which ranks are significantly different. The test results can be visualised in a critical distance plot. The critical distance describes the distance of mean ranks that two methods must have such that they are significantly different. Once it has been rejected that the mean of all method's ranks is equal, the Nemenyi Test[S11] can be performed to determine which ranks are significantly different. The test results can be portrayed in a critical distance plot. The critical distance describes the distance of mean ranks that two methods must have such that they are significantly different.

3.2 Results

The global performances on the external test set measured in RMSE is shown in Figure S2.

After performing the Friedman test, it was found that the hypothesis that all mean ranks are the same could be rejected. As such, Figure S1 shows the results of the Nemenyi test in critical distance plots, such that when methods are not statistically different in their mean rank, a bar is drawn between them. Three critical distance plots are drawn: the critical distance plot over experimental folds in Figure S1a, over target species in Figure S1b, and over compounds in Figure S1c. The equivalent results for the external validation are shown in Figure S3.

The methods MAML, fine-tuning all, multitask mean, multitask neural network (with multiple output nodes) and the single-task means are often statistically worse than other

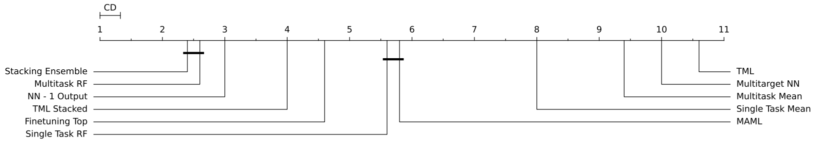

(a) Statistical significant differences in mean rank over experimental folds evaluated via the Nemenyi Test. The critical distance that must be between two mean ranks to be significantly different is 0.36.

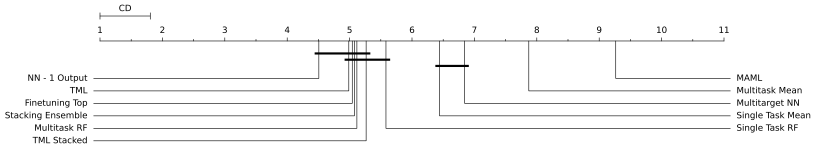

(b) Statistical significant differences in mean rank over target species evaluated via the Nemenyi Test. The critical distance that must be between two mean ranks to be significantly different is 0.81.

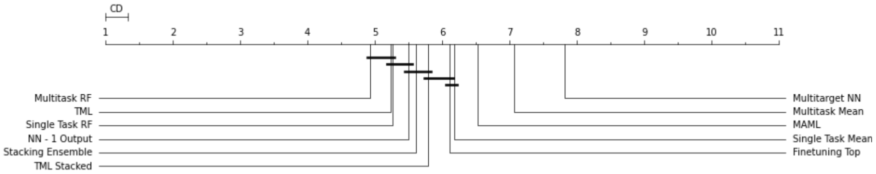

(c) Statistical significant differences in mean rank over chemicals evaluated via the Nemenyi Test. The critical distance that must be between two mean ranks to be significantly different is 0.33.

Figure S1: Critical distance plots for statistical differences of mean ranks in the *internal validation* with a significance value of $\alpha = 0.05$.

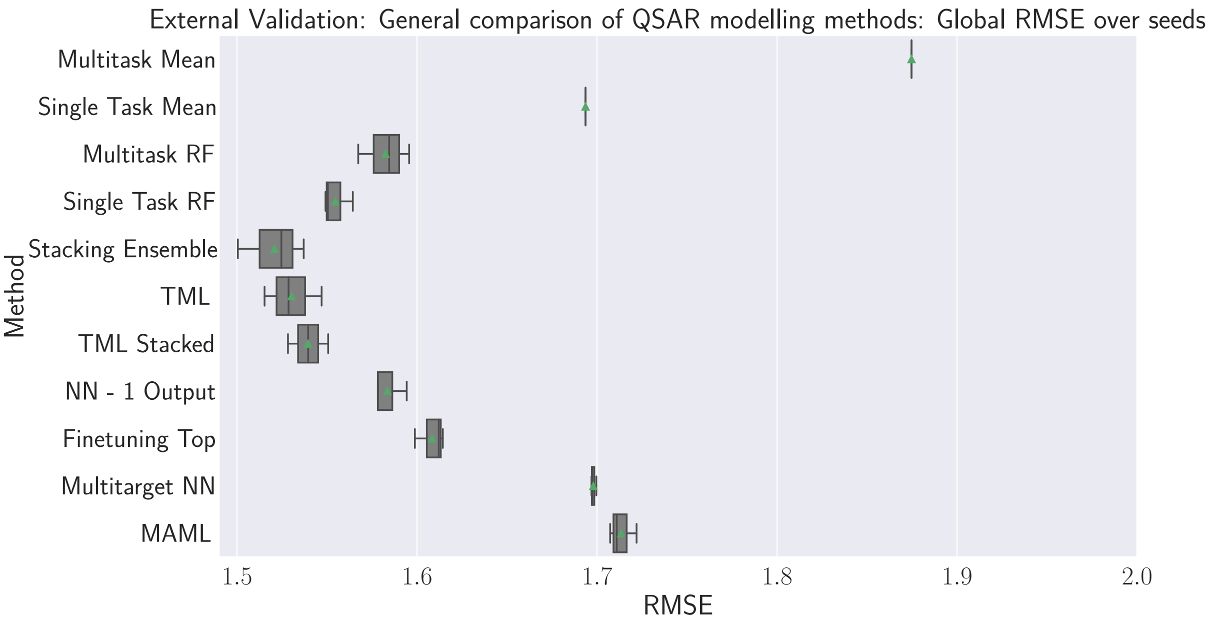

Figure S2: Comparison of Prediction Performances (RMSE) of different algorithms. The green marker shows the mean of the performances, whereas the line refers to the median performance.

methods. The best-performing methods in the general comparison so far have been the multitask neural network with one output node, the stacked ensembling method, and the multitask random forest. The neural network is found to be significantly worse than the multitask random forest when considering performances over experimental folds, and both the neural network and the stacked ensemble method are significantly worse than the multitask random forest when averaging performances over chemicals, whereas the multitask random forest is not found to be significantly worse than any other method in any critical distance plot diagram.

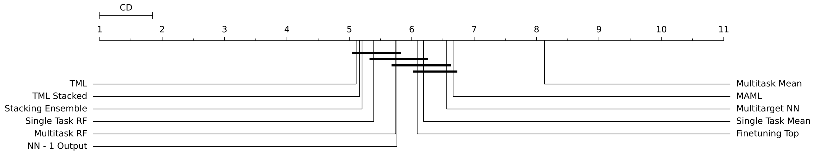

(a) Statistical significant differences in mean rank over target species evaluated via the Nemenyi Test. The critical distance that must be between two mean ranks to be significantly different is 0.84.

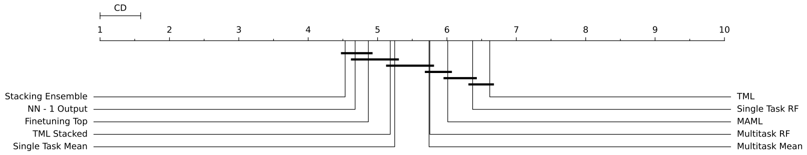

(b) Statistical significant differences in mean rank over chemicals evaluated via the Nemenyi Test. The critical distance that must be between two mean ranks to be significantly different is 0.59

Figure S3: Critical distance plots for statistical differences of mean ranks in the *external validation* with a significance value of $\alpha = 0.05$.

4 The influence of data sparsity in the learning curve performances

In the learning curve experiments investigating the prediction performance as a function of number of species, a performance difference was found to be due to a single chemical.

For this, the performance error of the multitask random forest at the anchor with 22 auxiliary species included in the dataset is analysed. Figure S4 gives the prediction error

for the chemical per species, whenever that chemical was in the test set of a species. All chemicals are included that were present in over 10 study species' test sets.

The Figure shows that the chemical represented by the SMILES *'ClC1(Cl)C(Cl)C(Cl)C(Cl)C1Cl'* is present in most of the test sets and has a particularly high error for most of these species. The chemical is present in 63 species, which is very high compared to other chemicals. Its high error rate shows that it is particularly difficult to predict.

As we have a sparse dataset, the errors averaged over the species are influenced by the combined high error and high presence of the chemical. When averaging the prediction error of the chemicals, this chemical is given less weight and influences the learning curve less.

This chemical is not included in the learning curves that investigate the prediction performance as a function of number of sssays.

5 Performance on real low resource tasks

Although the learning curves already investigate to what extent modelling techniques are impacted by less data, it is important to note that these experiments were performed on artificially downsampled datasets. Specifically, to show learning curves with long sampling sequences, the species with the most smaples were downsampled. Using large datasets to model low-resource situations has two main issues: the way the datasets are downsampled imposes a bias, and the assumption that downsampled large datasets behave like low-resource datasets can be false.

To show why this assumption may be false, it is vital to take a look at why there are disparities in the number of toxicity values per species. Eco-toxicity datasets are typically created on an ad-hoc basis, meaning that chemicals are typically tested once their production value exceeds the threshold and the REACH ruling requires toxicity values for the chemicals to be provided. For this, toxicity measures for daphnia, algae, and fish are required, although no exact species of these groupings is dictated.[S12] Thus, it is typical that testing facilities

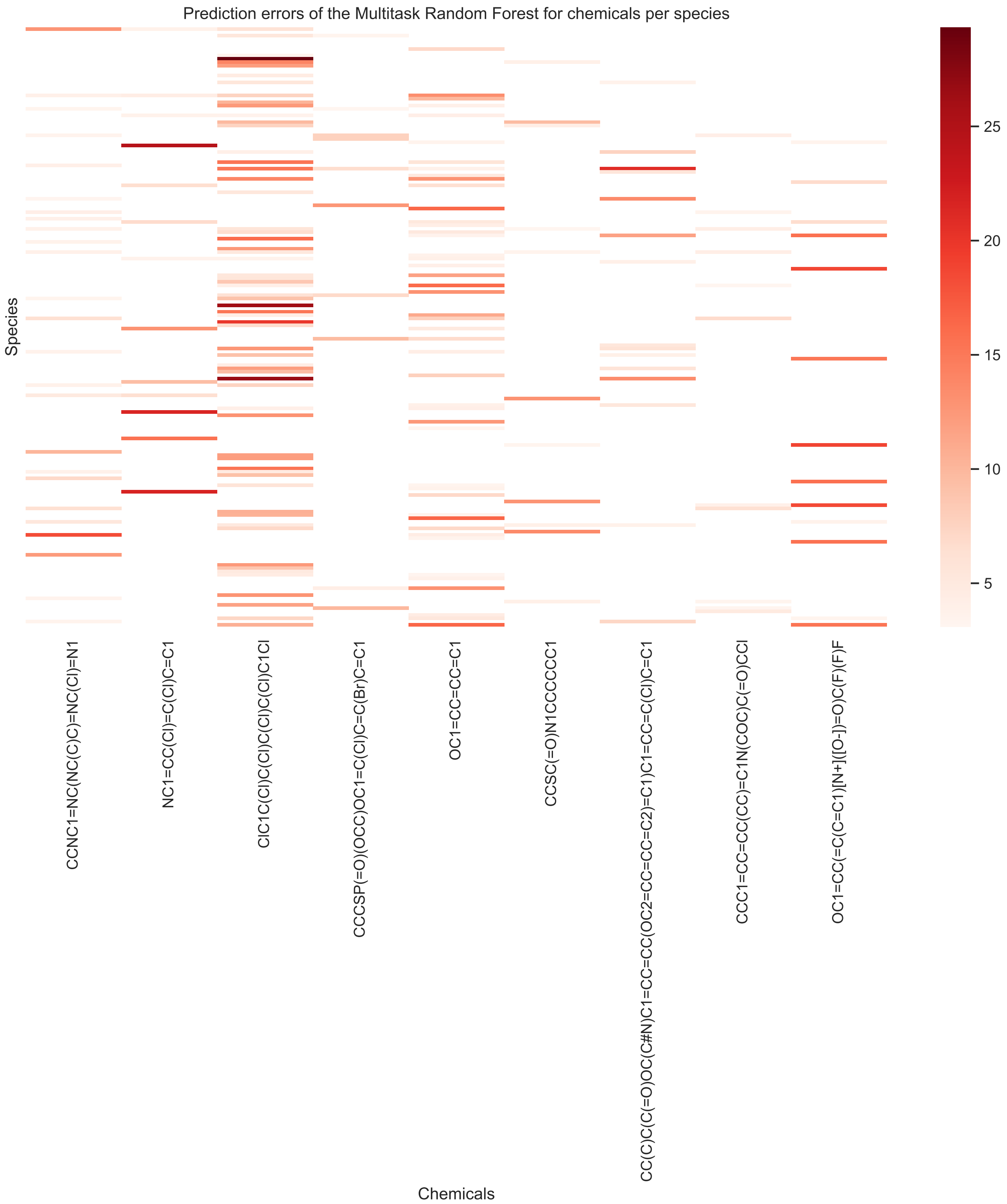

Figure S4: A heatmap of prediction errors of the multitask random forest for a chemical per species

use low-cost - in terms of time, expense and effort - species to acquire toxicity values. This leads to collections of toxicity datasets in species having a large variance in the number of samples: low-cost species have many samples, whereas species that are tested for a specific use case only are only available in small samples. Since there is a certain selection bias when choosing the species to test chemicals on, there is reason to believe that modelling of downsampled and low-resource datasets can not be assumed to behave equally.

Hence, we focus on real low-resource datasets and whether the trend found in the learning curves can be seen here, too. For this, the external validation of the QSAR models is taken, which sees the models training on 80% of the data and being tested on an external test set. We observe the datasets that have less than a certain number of compounds in their training set.

This section takes a look at the performances of actual low-resource datasets that are predicted using the complete ECOTOX training set as auxiliary data. Only the methods that have been interesting so far and the mean baselines for comparison are shown in Figure S5. It can be seen that although the multitask mean is a decent predictor when merely one or two compounds have been seen for a species, it quickly is outperformed by the other models once more compounds have been seen. As observed in the sampled learning curve with auxiliary data, the fine-tuning top method performs very well with extremely low-resource datasets. Fine-tuning the neural network when only one compound has been seen so far seems to calibrate the neural network well, such that performance improves by over 1 RMSE. Once more samples are added, the fine-tuning top is soon outperformed by the neural network.

The single-task mean and random forest are very similar in their average ranks and continuously improve as more compounds are added to the training set. Generally, it can be seen that the methods' performances on low-resource datasets are rather noisy and conclusions should be drawn with caution. As such, towards higher compound counts, the ranks of the separate methods seem to grow more similar.

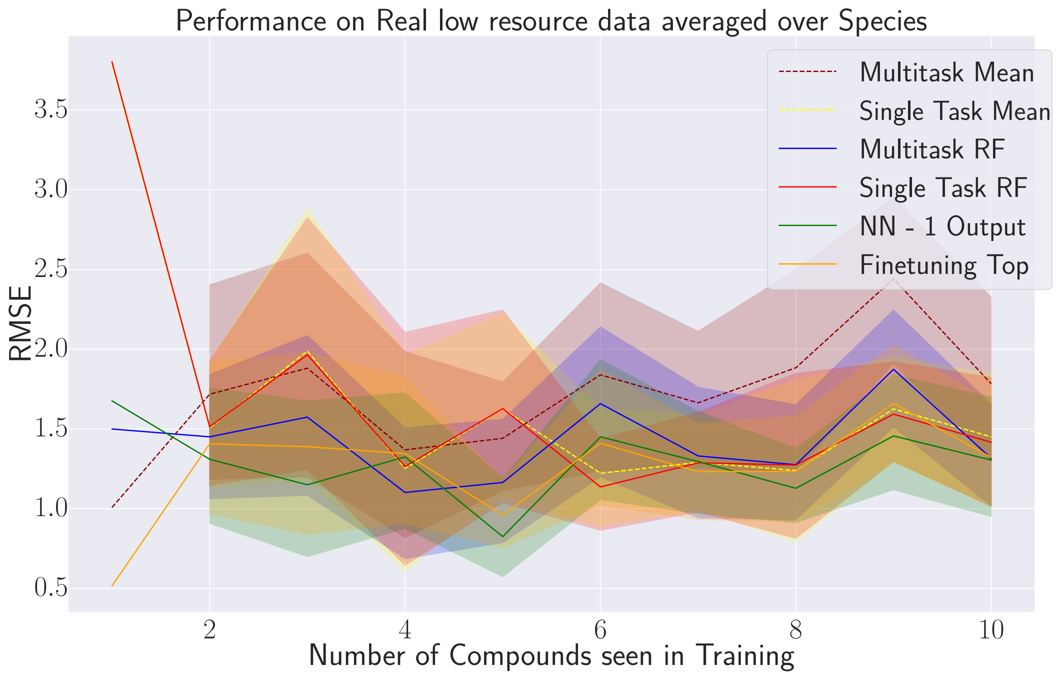

(a) RMSE Performances averaged over species for different compound counts seen.

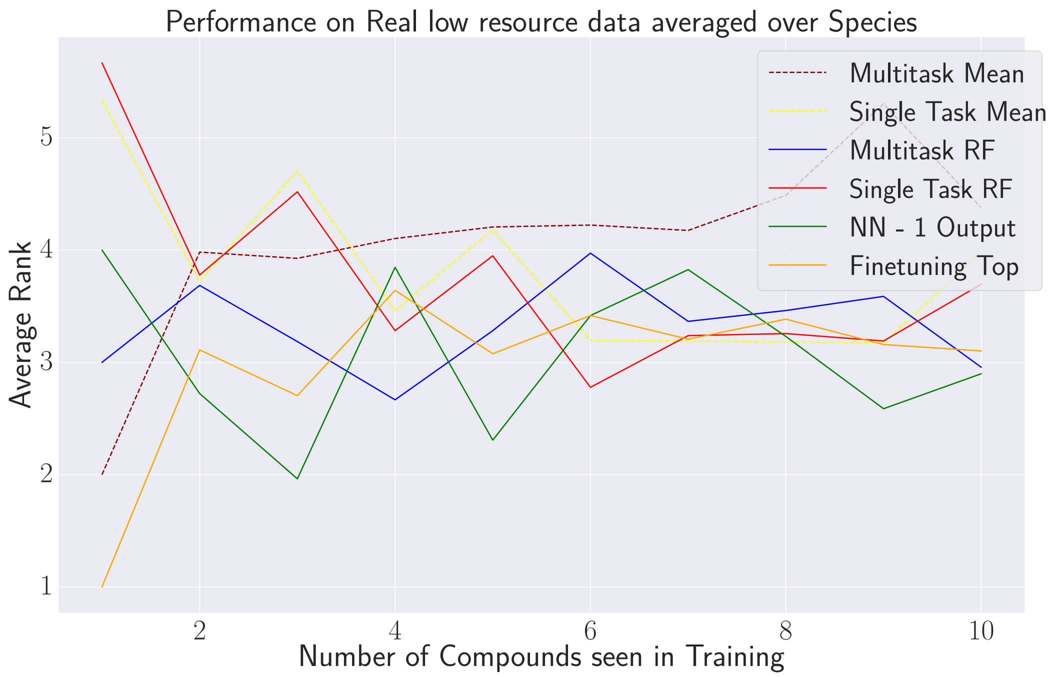

(b) Average ranks averaged over species for different compound counts seen.

Figure S5: Prediction performances on actual low-resource datasets.

6 Data

Table S8 gives an overview of all features in the gathered dataset. For further information on the species and chemicals used, as well as the dataset in its entirety please refer to our Git-Repository.[S13]

Feature Name	Feature Description
ECOTOX gathered data	
CAS Number	Chemical Abstracts Registration number
Test organisms (species)	Tested aquatic organism species name
Superclass	Tested aquatic organism species superclass
Phylum	Tested aquatic organism species phylum
Class	Tested aquatic organism species class
Duration.MeanValue	test duration in days
Value.MeanValue	LC50 or EC50-immobilization, in log(mg/l). The average value of the logarithmic value has been calculated (geometric mean of the non-log transformed values) for test results for the same species with the same test duration, in order to avoid model training bias towards chemicals that have been tested (in the same species) many times.
SMILES	The unique SMILES code representing the (2-dimensional) chemical structure. This SMILES-code representation of the chemical structure has been used to generate/predict to following structure/phys-chem properties, as well as the chemical fingerprint.
MolWeight	The molecular weight (g/mol) of the chemical structure represented by the SMILES-code (column I)
PaDEL generated descriptors	

nbAtoms	number of atoms (total) in the SMILES representation of the chemical structure, including the hydrogen atoms.
nbHeavyAtoms	number of 'heavy' atoms (total) in the SMILES representation of the chemical structure, i.e. not counting the hydrogen atoms
nbC	number of Carbon atoms in the SMILES representation of the chemical structure
nbO	number of Oxygen atoms in the SMILES representation of the chemical structure
nbN	number of Nitrogen atoms in the SMILES representation of the chemical structure
nbAromAtom	number of aromatic atoms in t he SMILES representation of the chemical structure
nbRotBd	number of rotatable bonds in the SMILES representation of the chemical structure
nbHBdAcc	number of Hydrogen bond Acceptors in the SMILES representation of the chemical structure
ndHBdDon	number of Hydrogen bond Donors in the SMILES representation of the chemical structure
ionization	ionization (0=neutral, 1=positively charged, 2=negatively charged)
nbRingimputed	number of ring systems in the SMILES representation of the chemical structure
nbHeteroRingimputed	number of hetero-ring systems in the SMILES representation of the chemical strcuture
TopoPolSurfAirimputed	Topological Polar Surface Area

MolarRefractimputed	Molar Refractivity
CombDipolPolarizimputed	Combined dipolarity/polarizability of the chemical structure represented by the SMILES (unit: dipole moment/ volume)
LogP_predimputed	estimate of the 10log of the octanol-water partition coefficient
LogVP_predimputed	estimate of the 10log of the vapour presssure (Pa)
LogWS_predimputed	estimate of the 10log of the water solubility (mg/L)
LogKOA_predimputed	estimate of the 10log of the octanol-air partition coefficient
LogD55_predimputed	estimate of the 10log pH-dependent octanol-water coefficient (log D) at pH 5.5
LogD74_predimputed	estimate of the 10 log of the pH dependent octanol-water coefficient (log D) at pH 7.4
Our added features	
missing_indicator_binary	Missing value indicator

Table S8: Overview of all features in the gathered dataset.

References

(S1) Feurer, M.; Klein, A.; Eggensperger, J., Katharina Springenberg; Blum, M.; Hutter, F. Efficient and robust automated machine learning. Advances in Neural Information Processing Systems 28. 2015; pp 2962–2970.

(S2) Srivastava, N.; Hinton, G.; Krizhevsky, A.; Sutskever, I.; Salakhutdinov, R. Dropout: a simple way to prevent neural networks from overfitting. *The Journal of Machine Learning Research* **2014**, *15 (1)*, 1929–1958.

(S3) Ioffe, S.; Szegedy, C. Batch normalization: accelerating deep network training by reducing internal covariate shift. International conference on machine learning. 2015; pp 448–456.

(S4) Microsoft, Neural Network Intelligence. 2021; `https://github.com/microsoft/nni`, Accessed 2023-05-19.

(S5) Paszke, A.; Gross, S.; Massa, F.; Lerer, A.; Bradbury, J.; Chanan, G.; Killeen, T.; Lin, Z.; Gimelshein, N.; Antiga, L.; Desmaison, A.; Köpf, A.; Yang, E. Z.; DeVito, Z.; Raison, M.; Tejani, A.; Chilamkurthy, S.; Steiner, B.; Fang, L.; Bai, J.; Chintala, S. PyTorch: An Imperative Style, High-Performance Deep Learning Library. Advances in Neural Information Processing Systems 32. 2019; pp 8024–8035.

(S6) Bergstra, J.; Bardenet, R.; Bengio, Y.; Kégl, B. Algorithms for hyper-parameter optimization. 2011.

(S7) Kingma, D. P.; Ba, J. Adam: a method for stochastic optimization. International Conference on Learning Representations. 2015.

(S8) Ramsundar, B.; Eastman, P.; Walters, P.; Pande, V.; Leswing, K.; Wu, Z. *Deep Learning for the Life Sciences: Applying Deep Learning to Genomics, Microscopy, Drug Discovery, and More*; O'Reilly Media, 2019.

(S9) Demšar, J. Statistical Comparisons of Classifiers over Multiple Data Sets. *The Journal of Machine Learning Research* **2006**, *7*, 1–30.

(S10) Friedman, M. A comparison of alternative tests of significance for the problem of m rankings. *The Annals of Mathematical Statistics* **1940**, *11 (1)*, 86–92.

(S11) Nemenyi, P. B. *Distribution-free multiple comparisons.*; Princeton University, 1963.

(S12) Lunghini, F.; Marcou, G.; Azam, P.; Enrici, M.; Van Miert, E.; Varnek, A. Consensus QSAR models estimating acute toxicity to aquatic organisms from different trophic levels: algae, Daphnia and fish. *SAR and QSAR in Environmental Research* **2020**, *31 (9)*, 655–675.

(S13) Schlender, T. Code Repository of "The Bigger Fish - Aquatic Toxicity QSAR models". `https://github.com/ADA-research/TheBiggerFish`.
